# Vancomycin-induced gut microbial dysbiosis alters enteric neuron–macrophage interactions during a critical period of postnatal development

**DOI:** 10.3389/fimmu.2023.1268909

**Published:** 2023-10-12

**Authors:** Ellen Merrick Schill, Elisabeth L. Joyce, Alexandria N. Floyd, Sreeram Udayan, Brigida Rusconi, Shreya Gaddipati, Bibiana E. Barrios, Vini John, Mitchell E. Kaye, Devesha H. Kulkarni, Jocelyn T. Pauta, Keely G. McDonald, Rodney D. Newberry

**Affiliations:** ^1^Division of Gastroenterology, Department of Medicine, Washington University School of Medicine, St. Louis, MO, United States; ^2^Division of Newborn Medicine, Department of Pediatrics, Washington University School of Medicine, St. Louis, MO, United States; ^3^Division of Gastroenterology, Hepatology and Nutrition, Department of Pediatrics, Washington University School of Medicine, St. Louis, MO, United States

**Keywords:** neonatal dysbiosis, muscularis macrophage, enteric nervous system, early life antibiotics, monocyte recruitment

## Abstract

Vancomycin is a broad-spectrum antibiotic widely used in cases of suspected sepsis in premature neonates. While appropriate and potentially lifesaving in this setting, early-life antibiotic exposure alters the developing microbiome and is associated with an increased risk of deadly complications, including late-onset sepsis (LOS) and necrotizing enterocolitis (NEC). Recent studies show that neonatal vancomycin treatment disrupts postnatal enteric nervous system (ENS) development in mouse pups, which is in part dependent upon neuroimmune interactions. This suggests that early-life antibiotic exposure could disrupt these interactions in the neonatal gut. Notably, a subset of tissue-resident intestinal macrophages, muscularis macrophages, has been identified as important contributors to the development of postnatal ENS. We hypothesized that vancomycin-induced neonatal dysbiosis impacts postnatal ENS development through its effects on macrophages. Using a mouse model, we found that exposure to vancomycin in the first 10 days of life, but not in adult mice, resulted in an expansion of pro-inflammatory colonic macrophages by increasing the recruitment of bone-marrow-derived macrophages. Single-cell RNA sequencing of neonatal colonic macrophages revealed that early-life vancomycin exposure was associated with an increase in immature and inflammatory macrophages, consistent with an influx of circulating monocytes differentiating into macrophages. Lineage tracing confirmed that vancomycin significantly increased the non-yolk-sac-derived macrophage population. Consistent with these results, early-life vancomycin exposure did not expand the colonic macrophage population nor decrease enteric neuron density in CCR2-deficient mice. Collectively, these findings demonstrate that early-life vancomycin exposure alters macrophage number and phenotypes in distinct ways compared with vancomycin exposure in adult mice and results in altered ENS development.

## 1 Introduction

Given the risk of perinatally acquired infections, exposure to broad-spectrum antibiotics in the first weeks of life is common in human neonates, especially premature neonates (1, 2). While this antimicrobial treatment is lifesaving in the acute period, early-life antibiotic exposure profoundly alters the trajectory of the developing microbiome and the development of the intestinal immune system (3–6). While antibiotic exposure impacts gut homeostasis and the immune system in adult mice, these effects are relatively transient compared with the effects of gut microbiota disruption during the critical neonatal period which can induce long-lasting effects on the gut and the immune system and risks of disease later in life (7–10). These changes in intestinal homeostasis induced by early-life antibiotic exposure increase the risks of complications of prematurity, necrotizing enterocolitis (NEC), late-onset sepsis (LOS), and later-life diseases such as food allergy and inflammatory bowel disease (11–15). Understanding how early-life antibiotics and changes to the developing microbiota alter intestinal immune development could identify novel strategies to mitigate the risk of these complications while preserving the benefits of antibiotic use in early life.

While the role of the microbiota in the development of the neonatal mucosal immune system is well established, only recently have studies identified a role for the microbiota in the developing nervous system of the gut, the enteric nervous system (ENS) (7, 16). The ENS is a branch of the autonomic nervous system that colonizes the length of the intestine and is made up of a network of neurons and glial cells (17). The ENS is organized into two separate but interconnected plexuses: the myenteric plexus with cell bodies located between the circular and longitudinal muscle layers of the bowel and the submucosal plexus located above the muscle layer and below the epithelium. ENS development begins in early gestation in both mice and humans but undergoes significant maturation (including subtype specification, electrophysiological signatures, and synapse formation) in the first weeks of postnatal life (17, 18). A recent work established that postnatal ENS development requires the neonatal microbiome. Germ-free mice, and neonatal mice treated with vancomycin, have decreased neuronal density, altered neuronal excitability, and disrupted intestinal motility both acutely and after weaning (19–21). These studies show that early-life disruption to the neonatal microbiota can have long-lasting effects on bowel homeostasis due to alterations in ENS development. Yet, how disruptions of the gut microbiota in early life mediate these events on ENS development is not well understood.

The ENS enacts effects on gut homeostasis through interactions with epithelial, muscle, and immune cells (22–26). A crosstalk between the ENS and a subtype of tissue-resident muscularis macrophages is well characterized in adult mice for its role in promoting motility and modulating macrophage phenotype. Recent studies have also identified important neuron/macrophage interactions in juvenile mice (27–29). While muscularis macrophages do not require an intact ENS for prenatal development, enteric neuron signaling reduces inflammatory signaling in intestinal macrophages in early postnatal life (30, 31). Conversely, muscularis macrophages are important in pruning the ENS in the first weeks of life in mouse models, demonstrating the requirement for muscularis macrophages in postnatal ENS development (27, 32). Understanding the role of macrophage–neuron interactions in early life is complicated by the rapid change in origin and phenotype of the colonic macrophage population in the first weeks of life. Colonic tissue-resident macrophages are predominantly yolk-sac-derived at birth but are almost entirely replaced and/or diluted out at the time of weaning by CX_3_CR1+ tissue-resident macrophages which are derived from circulating CCR2+ monocytes (33). Furthermore, the tissue-resident muscularis macrophages that interact most closely with the ENS in adulthood are an exception in that in homeostasis they are not rapidly replaced by bone marrow macrophages. Instead, resident muscularis macrophages are long-lived, self-replicating, and proposed to have origins as yolk-sac-derived macrophages (34). Muscularis macrophages and enteric neurons support each other via symbiotic secretion of growth factors. The enteric neurons secrete colony-stimulating factor 1 (CSF1), while the muscularis macrophages produce bone morphogen protein 2 (BMP2) (27). Notably, the CSF1/BMP2-mediated crosstalk between neurons and muscularis macrophages is microbiota-dependent. Adult mice treated with long-term broad-spectrum antibiotics have reduced levels of colonic BMP2 (27). Furthermore, the concentration of CSF1 and BMP2 increases over the preweaning period in congruence with the development of the gut microbiota (30). Given the importance of the microbiota in the maturation of the ENS and macrophages independently, we evaluated the unique and time-limited effects of early-life vancomycin treatment on colonic macrophage phenotype, enteric neuron/macrophage interactions, and ENS development.

In this study, we show that vancomycin, an antibiotic commonly used in neonatal intensive care units, induces intestinal macrophage expansion in neonatal mice but not in adult mice (2, 20). Employing single-cell RNA sequencing paired with fate mapping strategies for the first time in a neonatal dysbiosis model, we demonstrate that neonatal antibiotic exposure is associated with an expansion of pro-inflammatory macrophages in the colon due to a pronounced influx of bone-marrow-derived macrophages. The changes in macrophage numbers and phenotype were associated with disruption in the signaling and spatial relationships between the muscularis macrophages and the colonic enteric nervous system and increased phagocytosis of neurons. Given the expansion of bone-marrow-derived macrophages in vancomycin-treated pups, we assessed how C–C motif chemokine receptor 2 (CCR2) deficiency, which impairs recruitment of monocytes to the colon, affected these vancomycin-induced effects. Vancomycin did not significantly impact macrophage number or enteric neuron density in CCR2-deficient mice. These data suggest that disruptions to the developing neonatal microbiota by vancomycin alter the recruitment and phenotype of colonic macrophages in a CCR2-dependent manner during a critical period of development. These changes in macrophage number and phenotype have consequences for postnatal ENS and neuroimmune interactions.

## 2 Materials and methods

### 2.1 Mice

Mice used in these experiments were obtained from The Jackson Laboratory and then bred and maintained in-house. All strains were bred for at least 10 generations to the C57BL/6 background. Mouse strains used were wild-type C57BL/6 mice (catalog # 000664), Ai9 (Rosa26 tdTomato reporter mice, catalog # 007909) (35), CX_3_CR1^GFP^ mice (catalog # 005582) (36), CX_3_CR1^ERTCre^ mice (catalog # 020940) (37), BAF53b-Cre (catalog # 027826) (38), and CCR2^GFP^ mice (catalog # 027619) (39). Animal procedures and protocols were performed in accordance with the Institutional Animal Care and Use Committee at Washington University School of Medicine. Newborn mice were designated days of life (DOL) 0 animals. Neonatal male and female pups were used in ratios as naturally occurred in investigated litters. Due to known sex differences in the phenotype of adult mice treated with vancomycin as neonates, we selected to utilize only male mice in our adult experiments (21).

### 2.2 Vancomycin treatment

Litters of mice were randomly assigned to treatment groups. Neonatal mice were fed 83 mg/kg/day of vancomycin (GBiosciences, St. Louis, MO, USA) in deionized water orally via a pipette as previously described (20). Controls were fed water or were untreated. Mice were treated daily from DOL 1 to DOL 9/10 before analysis on DOL 10/11. Adult mice were placed on 0.5 g/L of vancomycin with 2 g/L of KoolAid or KoolAid alone in their drinking water for 10 days. This regimen has been shown to be equivalent in adult mice to oral feeding of 83 mg/kg/day in neonatal mice (40).

### 2.3 Tamoxifen treatment

CX_3_CR1^ERT+/−^ Ai9^+/−^ mouse pups were injected with 0.28 mg tamoxifen (Sigma-Aldrich, St. Louis, MO, USA) dissolved in sunflower oil i.p. once on DOL 1.

### 2.4 Isolation of cellular populations

Briefly, colons were isolated from appropriate animals and prepared as follows: Colons were opened along the mesenteric border (fat removed in adults), and the epithelial layer was removed via incubation at 37°C while shaking in Hank’s balanced salt solution (HBSS) (BioWhittaker, Walkersville, MD, USA) containing 5 mM of EDTA without Mg++ and Ca++ for 15 min in 50-ml falcon tubes at 37°C. Then, the tissue was cut into further small pieces and then digested using final concentrations of 0.2 U/ml of dispase (Fisher Scientific, Pittsburgh, PA, USA) and 25 U/ml of type VIII collagenase (Sigma) diluted in Roswell Park Memorial Institute medium (RPMI) (Gibco, Grand Island, NY, USA), 5% FCS at 37°C under shaking conditions for approximately 40 min. The digested tissue was then filtered through a 70-µm cell strainer, centrifuged at 1,500 rpm for 5 min, and washed before performing the flow cytometry analysis.

### 2.5 Flow cytometry

Single-cell suspensions were stained with antibodies against appropriate extracellular markers (**Supplementary Table 1**) in a buffer containing anti-CD16/32 antibodies to reduce non-specific signaling. Flow cytometry was performed on an Invitrogen Attune NXT cytometer. Gating was based on full minus one stained populations (**Supplementary Figure 2**).

### 2.6 Fluorescence-activated cell sorting and single-cell RNA-seq

Single-cell suspensions from the colons of the control and vancomycin-treated litters of CX_3_CR1^GFP^ heterozygous pups were pooled from the entire litter. Flow cytometric cell sorting was performed on a FACS Aria BD Fusion cell sorter. Appropriate cells were identified as live, CD45^+^ CD11c^lo/−^ CX_3_CR1^+^ and collected in supplemented RPMI 1640 media. RNA barcoding, library preparation, and sequencing were performed using the 10X Chromium Controller and 10X Genomics 3’ v3.1 platform through the McDonnell Genome Institute at the Genome Technology Access Center at Washington University in Saint Louis School of Medicine. Briefly, 3v3.1 cDNA was prepared after the GEM generation and barcoding, followed by the GEM-RT reaction and bead cleanup steps. Purified cDNA was amplified for 11–13 cycles before being cleaned up using SPRIselect beads. Samples were then run on a Bioanalyzer to determine the cDNA concentration. GEX libraries were prepared as recommended by the 10X Genomics Chromium Single Cell 3′ Reagent Kits User Guide (v3.1 Chemistry Dual Index) with appropriate modifications to the PCR cycles based on the calculated cDNA concentration. For sample preparation on the 10X Genomics platform, the Chromium Next GEM Single Cell 3′ Kit v3.1, 16 rxns (PN-1000268); Chromium Next GEM Chip G Single Cell Kit, 48 rxns (PN-1000120); and Dual Index Kit TT Set A, 96 rxns (PN-1000215) were used. The concentration of each library was accurately determined through qPCR utilizing the KAPA library quantification kit according to the manufacturer’s protocol (Sigma-Aldrich, St. Louis MO, USA; Roche - Little Falls, NJ, USA) to produce cluster counts appropriate for the Illumina NovaSeq6000 instrument. Normalized libraries were sequenced on a NovaSeq6000 S4 Flow Cell using the XP workflow and a 50 × 10 × 16 × 150 sequencing recipe according to the manufacturer’s protocol. A median sequencing depth of 50,000 reads/cell was targeted for each Gene Expression Library. Data were analyzed using Partek Flow. Clusters identified as inconsistent with macrophages were excluded and cells were reclustered (**Supplementary Figure 4**, **Supplementary Table 4**; initial clusters 4, 8, 9, 10, and 11 were excluded from further analysis). Differentially expressed genes were defined by gene set analysis (GSA) with a false discovery rate of less than or equal to 0.05 and fold change of greater than 2 or less than −2. Trajectory and pseudotime analyses were performed with Monocle 3. The RNAseq dataset has been deposited and is publicly available on NCBI GEO (GSE245019).

### 2.7 Bacterial 16S sequencing and analysis

Bacterial DNA was isolated from colonic contents from DOL 10/11 pups or adults upon animal sacrifice. Stools were frozen at −20°C or −80°C until analysis. Stools from three to four pups per litter were pooled for neonatal analysis, and litters are the biological replicates. Bacterial DNA was isolated with the QIAamp Fast DNA Stool Mini kit. A library was generated and samples were barcoded with high fidelity Taq polymerase. PCR products were gel-purified and submitted for sequencing of the V4 regions of 16S ribosomal RNA. Briefly, the quality and concentration of the samples were assessed on an Agilent BioAnalyzer High Sensitivity DNA Chip. Samples were denatured and libraries were sequenced using the MiSeq 500v2 Illumina kit, and 2 × 250 sequencing depth was achieved on an Illumina MiSeq. QIIME2 version 2023.5 was used to perform quality control and analysis of the 16S V4 sequence reads (41). Raw fastq files were demultiplexed and denoised with dada2 (42). Taxonomy was assigned to the amplicon sequence variants (ASVs) using the feature classifier classify-consensus-blast against preformatted SILVA reference and taxonomy files (43, 44). MAFFT was used for multiple ASV sequence alignment via q2‐alignment and used to construct a phylogeny with fasttree2 via q2-phylogeny. Alpha diversity was measured using the Shannon (*H*) index. Beta diversity (community dissimilarity) metrics were computed with q2-diversity, including Bray–Curtis. Group comparisons of alpha diversity were performed with the Kruskal–Wallis test, and group comparisons of beta diversity were performed with non-parametric PERMANOVA (QIIME2). Differential abundance comparison between microbiome families between different treatment groups was performed by ANCOM (45). 16S rRNA sequencing data were submitted to the European Nucleotide Archive (ENA) available at project number PRJEB66081.

### 2.8 Immunostaining

Colons were pinned flat onto Sylgard plates in phosphate-buffered saline pH 7.2 (PBS), using minutien pins. The plate was then filled with 4% paraformaldehyde in PBS and left to sit for 45 min at room temperature. After fixation, the muscularis layer was peeled away from the submucosal layer of the colon via microdissection. The tissue samples were stored in 50% PBS/glycerol at −20° for later analysis. Samples were rinsed with PBS and then placed in a blocking buffer (PBS with 0.1% Tween-20, Fischer Scientific, and 1% bovine serum albumin, Fisher Scientific) at room temperature for 1 h. Samples were stained with primary antibody overnight at 4°C and with secondary antibody for 1 h at room temperature (see **Supplementary Table 1** for antibodies and concentrations). The samples were mounted on frosted glass slides (Fisher Scientific) with a glass coverslip (VWR) using 50% PBS/glycerol and stored at 4°C until image acquisition.

### 2.9 Imaging and data analysis

Immunostained tissues were imaged using a Zeiss widefield fluorescence microscope or Zeiss confocal microscope. For quantification of neuron or macrophage density, a minimum of 3 random images were taken from each tissue sample. Images were analyzed using FIJI ImageJ. Neuronal and macrophage cell bodies, per high-power field, were counted manually. The area covered by neuronal staining was measured by subtracting the background, then thresholding images, and recording the percentage of pixels covered by HuC/D staining. The average of the three images was used as a single data point per mouse. The length of macrophage projections was measured using the line tool on FIJI ImageJ. Quantification was measured per animal. The macrophage area was quantified in flattened confocal stacks via thresholding and region of interest identification.

### 2.10 Serotonin ELISA

Colonic contents were isolated at the time of pup sacrifice and frozen at −20°C or −80°C until analysis. Samples were thawed and 10 μl of PBS was added for every 1 mg of stool. Stools from two to four littermates were combined and litters were assessed as the biological replicate. Enzyme-linked immunosorbent assay (ELISA) was performed per kit instructions (Serotonin ELISA Kit, Abcam, Boston, MA, USA).

### 2.11 BMP2 and CSF1 ELISA

The whole colon (just distal to the cecum to the anus) from DOL 10 pups was homogenized in a solution of 200 ml of PBS/200 ml with radioimmunoprecipitation assay (RIPA) buffer with protease inhibitors. Samples were centrifuged and supernatants were frozen at −20°C until further processing. After thawing, samples were processed per kit instructions (BMP2 or CSF1 Quantikine ELISA Kit, R&D Systems, Minneapolis, MN, USA). A fixed volume of 50 μl was used for each sample and run in duplicate. Measurements represent BMP2 and CSF1 levels per milliliter of supernatant. Absorbance was measured on a BioTek Synergy HT Microplate Reader.

### 2.12 Data presentation and statistics

Data are presented as means ± standard deviation unless otherwise indicated. Each data point represents a unique biological replicate. Statistical comparisons between antibiotic-exposed mice and controls were performed using unpaired two-tailed *t*-test or one-way ANOVA (unless otherwise indicated for non-normally distributed data) with GraphPad Prism 9.0. P *<*0.05 was considered statistically significant.

## 3 Results

### 3.1 Vancomycin treatment expands the intestinal macrophage population in neonatal mice

**Figure 1:**
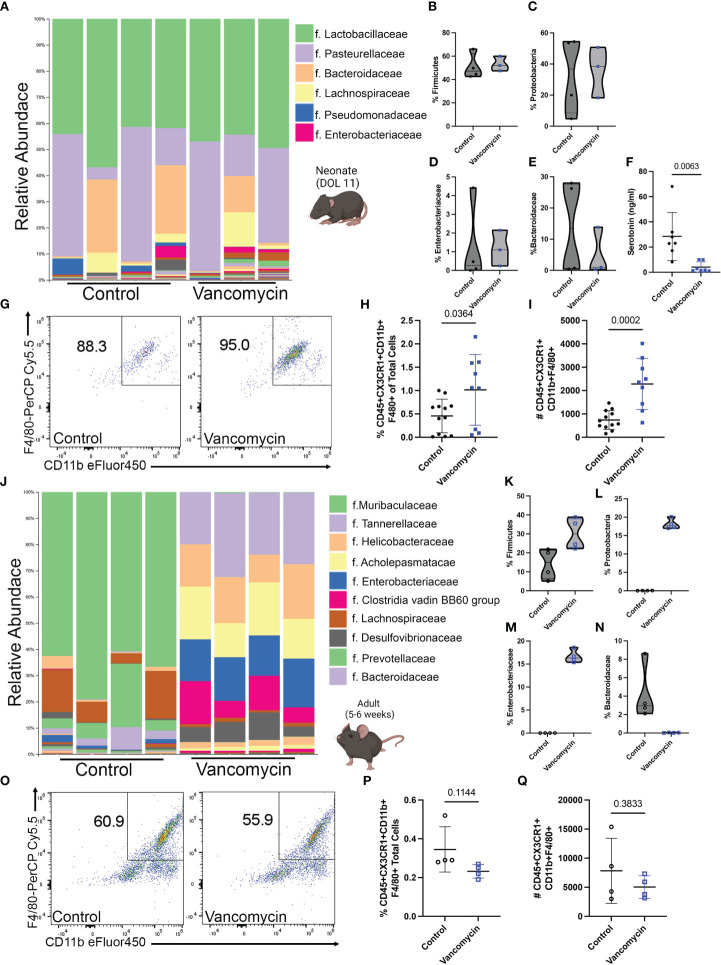
Vancomycin treatment expands the colonic macrophage population in neonatal but not adult mice. C57B6/J mouse pups were treated with 83 mg/kg/day of vancomycin orally from DOL 1 to 10/11 before analysis on DOL 10/11. **(A)** Taxonomic bar plots at the family level demonstrating the relative abundance of bacteria of control and vancomycin-treated litters. Relative abundance of bacteria in the **(B)** *Firmicute* [data presented as median (25–75 percentile); control 47.25 (43.07–62.06)%, *n* = 4 litters, 3–4 pups per litter; vancomycin 52.08 (47.45–59.99)%, *n* = 3 litters, 3–4 pups per litter] and **(C)** *Proteobacteria* [control 36.77 (8.59–54.18)%, vancomycin 38.54 (18.27–50.73)%] phyla. Relative abundance of bacteria in the **(D)** *Enterobacteriaceae* [control 0.27 (0.021–3.43)%, vancomycin 1.1 (0.24–2.15)%] and **(E)** *Bacteroidaceae* [control 13.5 (0.53–25.57)%, vancomycin 0.99 (0.27–13.84)%] families. *P*-value = 0.63 for all four comparisons by the Mann–Whitney test. **(F)** Serotonin concentration in colonic content as measured by ELISA was significantly reduced by vancomycin treatment (data presented as mean ± standard deviation): control 28.5 ± 19.1 ng/ml, *n* = 7 litters, 3–4 pups per litter; vancomycin 4.1 ± 4.1 ng/ml, *n* = 7 litters, 2–4 pups per litter. **(G)** Representative density plots of singlet CD45^+^ CX_3_CR1^+^ cells gated for CD11b and F4/80 on control and vancomycin-treated mice, respectively. Vancomycin increased the population of CD45^+^ CX_3_CR1^+^ CD11b- F4/80^+^ or macrophages both by the **(H)** percentage of total cells in the colon (control 0.45% ± 0.35%, *n* = 12; vancomycin 1.02% ± 0.75%, *n* = 9) and **(I)** absolute cell numbers (control 740 ± 404.7 cells, *n* = 12; vancomycin 2,283 ± 1,093 cells, *n* = 9). In comparison, adult male mice were treated with 0.5 g/L of vancomycin in their drinking water for 10 days prior to analysis. **(J)** Taxonomic bar plots at the family level demonstrating the relative abundance of bacteria from control or vancomycin-treated adult male mice. Relative abundance of bacteria in the **(K)** *Firmicute* [data presented as median (25–75 percentile); control 14.96 (6.29–21.45)%, *n* = 4; vancomycin 29.99 (22.81–37.98)%, *n* = 4] and **(L)** *Proteobacteria* [control 0.04 (0.01–0.05)%, vancomycin 17.7 (17.05–19.59)%] phyla. Relative abundance of bacteria in the **(M)** *Enterobacteriaceae* [control 0.02 (0.004–0.03)%, vancomycin 16.32 (5.52–18.11)%] and **(N)** *Bacteroidaceae* [control 2.97 (2.27–7.26)%, vancomycin 0.03 (0.005–0.05)%] families. *P*-value = 0.03 for all four comparisons by the Mann–Whitney test. **(O)** Representative density plots of singlet CD45^+^ CX_3_CR1^+^ cells gated for CD11b and F4/80 on adult control and vancomycin-treated mice, respectively. There was no difference in the **(P)** percentage (data presented as mean ± standard deviation; control 0.35% ± 0.12%, *n* = 4; vancomycin 0.23% ± 0.04%, *n* = 4) or **(Q)** absolute number (control 7,819 ± 5,586, *n* = 4; vancomycin 5,038 ± 1,939, *n* = 4) of macrophages between control and vancomycin-treated mice.

To determine the effect of early-life vancomycin treatment on neonatal intestinal macrophages, we treated wild-type mouse pups with vancomycin or water daily by gavage from day of life (DOL) 1–10/11 and sacrificed mice for analysis. We assessed how vancomycin treatment altered the neonatal intestinal microbiota by performing 16S V4 sequencing on bacterial DNA isolated from pooled litters of pup colonic contents. Overall, the neonatal microbiota had low diversity and were dominated by bacteria in the *Lactobacillaceae* and *Pasteurellaceae* families (**Figure 1A**). There were no statistically significant changes in alpha or beta diversity between the control and vancomycin-treated groups (**Supplementary Figures 1A, B**). Analysis of differential abundance by ANCOM did not identify differently abundant bacterial taxa in vancomycin-treated mice. Consistent with previous studies, vancomycin-treated mice had a modest expansion of bacteria in the *Firmicute* phylum, but we did not observe a concomitant decrease in the *Proteobacteria* phylum (**Figures 1B, C**) (20, 46). The microbiota of vancomycin-treated pups did have similarities with dysbiotic human neonatal microbiota (47–49). Vancomycin-treated mice had a trend of higher abundance of bacteria in the *Enterobacteriaceae* family and lower abundance of *Bacteroidaceae* although this did not reach statistical significance (**Figures 1D, E**). These data show that despite the low diversity found at this age in neonatal mice, vancomycin treatment does promote some degree of changes in the gut microbiota.

Despite the lack of significant change in the neonatal microbial taxa due to vancomycin treatment, we hypothesized that the microbial metabolome may be impacted. Previous studies have shown that the neonatal microbial metabolome is enriched in neurotransmitters and vancomycin treatment specifically has been shown to reduce the level of serotonin in neonatal colonic mucosa (20, 50). To determine if vancomycin treatment altered microbiota-derived serotonin in neonates, we measured serotonin levels in the colonic content of the control and vancomycin-treated P10/11 pups by ELISA. Vancomycin treatment significantly reduced luminal serotonin concentration compared with control mice (**Figure 1F**). These data show that despite only inducing small changes on the taxonomic level that can be assigned by V4 16S rRNA sequencing, vancomycin treatment disrupts the neonatal microbial metabolome.

### 3.2 Vancomycin treatment does not impact the intestinal macrophage population in adult mice

We next investigated how these changes in the microbiota impacted the neonatal mucosal immune system. CD45^+^ CX_3_CR1^+^ F4/80^+^ CD11b^+^ cells (colonic macrophages) were evaluated by flow cytometry (**Figure 1G**; **Supplementary Figure 2A**). As previously shown in neonatal germ-free mice, vancomycin-treated mice had an expansion of colonic macrophages compared with controls as quantified by both the percentage of total cells in the colon and the absolute number of cells (**Figures 1H, I**). Preweaning colonic macrophages are commonly characterized as being yolk-sac-derived or bone-marrow-derived based on their relative expression of F4/80 and CD11b. Yolk-sac-derived macrophages express F4/80^hi^ and bone-marrow-derived macrophages express a CD11b^hi^ signature (33). Neonatal germ-free mice have previously been demonstrated to have an expansion of F4/80^hi^ macrophages consistent with a yolk sac origin (51). To assess if vancomycin preferentially expanded the yolk-sac-derived colonic macrophage population, we quantified the percentage of F4/80^hi^ CD11b^lo^ colonic macrophages (**Supplementary Figure 3A**). There was no difference in the percentage of F4/80^hi^ CD11b^lo^ colonic macrophages (**Supplementary Figure 3B**). These data demonstrate that the subtle changes observed in the neonatal microbiota induced by vancomycin treatment affect the expansion of colonic macrophages via a different process than what has been observed in germ-free mice in the critical preweaning window.

As previous studies have identified that the preweaning interval is a critical period in which the mucosal immune system can be uniquely influenced by the microbiota, we then asked if vancomycin treatment in adults altered colonic macrophage quantity and phenotype. Five- to 6-week-old male mice were treated with vancomycin in their drinking water for 10 days. We assessed the impact of vancomycin on the adult microbiome with V4 16S rRNA sequencing of bacterial DNA isolated from colonic content as performed above in neonatal mice. Vancomycin-treated adult mice had a significantly altered microbiota as visualized by the taxonomy plot and confirmed with significant differences in alpha and beta diversity between the control and vancomycin-treated mice (**Figure 1J**; **Supplementary Figures 1C, D**). Notably, adult male mice treated with vancomycin have an increase in both *Firmicutes* and *Proteobacteria* at the phylum level compared with controls (**Figures 1K, L**). Furthermore, ANCOM analysis revealed that the vancomycin-treated adults have a significantly increased abundance of *Enterobacteriaceae* and a decreased abundance of *Bacteroidaceae*, demonstrating the same dysbiosis signature seen in neonatal mice (**Supplementary Table 2**; **Figures 1D, E, M, N**). Notably, postweaning treatment with vancomycin has previously been shown to have no effect on colonic enteric neuronal density or mucosal serotonin levels (52).

We next assessed how adult vancomycin treatment and its associated dysbiotic changes in the microbiota alter colonic macrophages using flow cytometry (**Figure 1O** showing F4/80 and CD11b gating on macrophages, following the gating strategy in **Supplementary Figure 2A**). Vancomycin treatment had no impact on the number of colonic macrophages (CD45^+^ CX_3_CR1^+^ CD11b^+^ F4/80^+^ cells) by percentage of total colonic cells or absolute number (**Figures 1P, Q**). These data demonstrate that despite more dramatic shifts in the microbiota, vancomycin treatment in adults does not expand the population of colonic macrophages, in contrast to the effect observed in neonates.

### 3.3 CX_3_CR1^+^ macrophages show increased expression of pro-inflammatory genes with vancomycin treatment of neonatal mice

**Figure 2:**
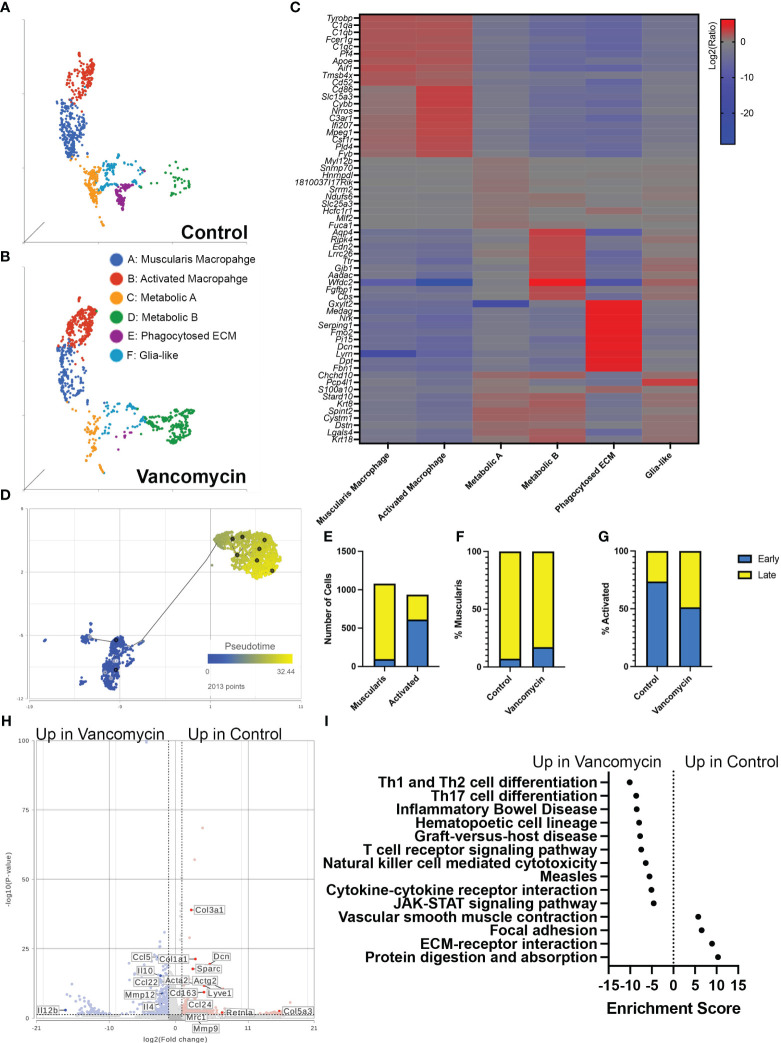
Vancomycin promotes the expression of pro-inflammatory genes in neonatal colonic macrophages. Clustering of CD45^+^ CX_3_CR1^+^ CD11c^−/lo^ cells from control **(A)** and vancomycin-treated **(B)** DOL 11 CX_3_CR1^GFP^ heterozygous mice based on downsampled counts to visualize the relative abundance of cell clusters between conditions. **(C)** Heatmap displaying the top 10 biomarkers visualized by the log2 ratio between the expression of the target cluster compared with the expression in all other clusters. **(D)** The muscularis and activated macrophage clusters were subsequently assessed by pseudotime and visualized per two-dimension UMAP plot with a place in pseudotime denoted by color from early (blue) to late (yellow). **(E)** Quantification of cells identified as early compared with late on trajectory analysis demonstrated a significant increase in early cells in the activated cohort compared with the muscularis cluster (*P* < 0.0001, chi-square test). Percentage of early and late cells in the **(F)** muscularis (control: early 7.18%, late 92.82%; vancomycin: early 17.24%, late 82.76%) and **(G)** activated cells (Control: early 73.65% late 26.35%; Vancomycin: early 51.31% late 48.69%) was altered by vancomycin treatment with a greater percentage of muscularis cells coded as early in the vancomycin-treated group and an increase in activated cells coded as late in the vancomycin-treated group. **(H)** Volcano plot demonstrating differential gene expression between control and vancomycin-treated colons of the pooled population of muscularis and activated macrophages. **(I)** KEGG gene expression pathways upregulated in vancomycin treatment (negative enrichment scores) compared with control (positive enrichment scores).

Our flow cytometry data suggested that the expansion of colonic macrophages observed in vancomycin-treated neonatal mice differs from what has been seen in germ-free mice (51). We further characterized the impact of early-life vancomycin exposure on colonic macrophages by assessing changes in the transcriptome using single-cell RNA sequencing. We isolated CD45^+^ CX_3_CR1^GFP+^ CD11c^lo/−^ cells using fluorescence-activated cell sorting from lamina propria preparations of pooled whole colons from litters of control or vancomycin-treated pups (**Supplementary Figure 4**). The exclusion of CD11c^+^ cells aimed to enrich the collected population in muscularis macrophages, the subpopulation of colonic resident macrophages identified to interact with the enteric nervous system (27, 32, 34, 53). After quality assessment, 3,380 cells from control mice and 1,528 cells from vancomycin-treated mice were included in the analysis. Unbiased clustering based on gene expression revealed 11 subsets, including cell types with gene expression patterns that were inconsistent with macrophages—likely acquired secondary to autofluorescence during sorting or CX_3_CR1 expression on non-macrophage subsets (**Supplementary Figure 5**, **Supplementary Table 3**) (54–56). These cell types were excluded, and the remaining cells were reclustered, identifying six distinct subtypes with unique biomarkers (**Figures 2A–C**, **Table 1**; **Supplementary Table 3**). We classified these six types utilizing available gene network algorithms (Metascape, WebGestalt, Human Protein Atlas) (57–59).

**Table 1:** Biomarkers of the six macrophage clusters identified by scRNA sequencing with relative abundance in control and vancomycin-treated cohorts.

	Muscularis macrophage	Activated macrophage	Metabolic A	Metabolic B	ECM phagocytosis	Glia-like
**Biomarkers**	Tyrobp, C1qa, C1qb, Fcer1g, C1qc, Apoe, Aif1, Tmsb4x, Cd52, Atp6v0e, Nmt1, Srsf9, Lamtor4, Wdr83os, Akr1b3, Erp20, Cenpx, Pitpna, Pomp	Cd86, Slc15a3, Cybb, Nrros, C3ar1, Ifi207, Mpeg1, CSf1r, Pld4, Fyb, Prprc, Cd300a, Cd53, Evi2a, Fcgr1, Ms4a5c, Adgre1, Lrrc25, Fcgr3, Tm6sf1	Myl12b, Snrnp70, Hnrnpdl, 1810037I17Rik, Srrm2, Ndufs6, Slc25a3, Hcfc1r1, Mlf2, Fuca1, Mrps16, Eif3d, Hdgf, Polr2f, Mdh1, Eif2s2, Clk1, P4hb, Eif4b, Lgals4	Aqp4, Ripk4, Edn2, Lrrc26, Ttr, Gjb1, Aadac, Wfdc2, Fgfbp1, Cbs, Cyp4f40, Cftr, Clca3b, Cldn2, 5330417C22Rik, Klk1, D630039A03Rik, Kcnk1, Kcnn4, Foxa3	Gxylt2, Medag, Nrk, Serping, Fmo2, Pi15, Dcn, Lvrn, Dpt, Fbn1, Aebp1, Itm2a, Cpxm1, Clec3b, Mfap5, Cpz, Efemp1, Ccdc8-, Mmp16, Peg3	Chchd10, Pcp4l1, S100a10, Stard10, Krt8, Spint2, Cystm1, Dstn, Lgals4, Krt18, Car2, Krt19, Nedd4, Cldn7, 2200002D01Rik, Epcam, Ajap1, Gpx2, Ckmt1
**% Control**	30.16	20.36	17.37	5.57	14.82	11.76
**% Vancomycin**	20.63	34.86	8.03	28.35	0.81	7.42

Cluster A was labeled as muscularis macrophages based on the enrichment in genes (*Tyrobp*, *C1qa*, *C1qb*, *C1qc*, *Apoe*, *Pf4*, *Aif1*) previously identified to be expressed in muscularis macrophages in mouse and human sequencing experiments (60, 61). Cluster B was labeled activated macrophages as this cluster was notable for its enrichment in markers of intestinal macrophages (*Csf1r*, *Adgre1*, *Fcgr1*) and inflammatory markers (*Ptprc*, *Cd86*, *Ifi207*, *Cybb*, *Nrros*, *Cd68*). Notably, there was a greater representation of the muscularis macrophage cluster in control compared with vancomycin-treated mice (30.6% vs. 20.63%), whereas the activated macrophage cluster was more abundant in the vancomycin-treated mice (20.36% vs. 34.86%). Clusters C (*Fuca1*, *Mdh1*, *Slc25a*) and D (*Aadac*, *Cbs*, *Cyp4f40*) were labeled as metabolic A and B given the upregulation of metabolic and oxidative pathways (**Supplementary Figure 6**). Despite a similar phenotype, the metabolic A and B clusters had differential abundance in the control and vancomycin-treated mice. Metabolic A represented 17.4% of CX3CR1^+^ macrophage in control mice and only 8% in vancomycin-treated mice. Conversely, metabolic B made up 5.6% of CX3CR1^+^ macrophages in control mice and 23.4% in vancomycin-treated mice. These changes in abundance may suggest alterations in intestinal immunometabolism induced by neonatal vancomycin treatment. Cluster E had enrichment in extracellular matrix (ECM) genes (*Gxylt2*, *Dcn*, *Serping*, *Fbn1*) consistent with a previously seen subset of neonatal muscularis macrophages thought to have this gene signature secondary to phagocytosis of fibroblast-like cells and was therefore labeled as phagocytosed ECM (32). Finally, cluster F was named glia-like with expression of genes associated with peripheral and enteric glia (*Chchd10*, *S100a10*, *Sox10*). Differential gene expression of identified biomarkers (**Table 1**) between each cluster was quantified by GSA based on log2 of the gene expression ratio in the target cluster compared with its expression in all the other clusters (**Figure 2C**). For the remainder of this paper, we focus on clusters A and B as the genes enriched in these subsets closely reflected classic intestinal macrophages and matched the cells assessed by flow cytometry in **Figure 1**.

Given the overlap in the muscularis macrophage and activated macrophage populations on UMAP plots, gene expression per KEGG pathway analysis, and differential gene expression (**Figures 2A–C**), we next aimed to further discriminate the differences between these two clusters. We hypothesized that these clusters may represent muscularis and mucosal macrophages (despite concentrating muscularis macrophages by sorting out CD11c^hi^ cells). We compared the genes enriched in these clusters to published lists from adult muscularis and mucosal macrophages (60). Our muscularis and activated macrophage clusters expressed genes found both in muscularis and mucosal macrophages in the other studies, with the activated cluster having more homology with both subtypes (**Supplementary Figure 7A**). We performed pathway analysis on genes found to be most differentially expressed between the two clusters based on GSA. The activated macrophage cluster showed preferential enrichment in pro-inflammatory pathways including TNF, Toll-like receptor, and NF-kappa B signaling pathways, whereas the muscularis macrophage cluster had enrichment in the vascular smooth muscle contraction (**Supplementary Figure 7B**). Differential gene expression between the two clusters visualized on a volcano plot also showed that the activated macrophage cluster had higher expression of genes associated with recently recruited monocytes maturing into macrophages (*Ccr2*, *Ly6c2*), supporting the enrichment of the monocyte differentiation pathway seen in the activated cluster (**Supplementary Figure 7A**). We then hypothesized that the two macrophage clusters were of shared origin but at different stages in their maturation. We performed trajectory analysis and found that activated macrophages occurred earlier in pseudotime (i.e., were more immature) compared with the muscularis macrophage cluster (**Figures 2D, E**). When we assessed maturation by treatment, we determined that vancomycin-treated muscularis macrophages (overall more mature on pseudotime analysis, **Figure 2D**) were more likely to be earlier in pseudotime analysis compared with control (**Figure 2F**), whereas vancomycin-treated activated macrophages (overall less mature on pseudotime analysis, **Figure 2D**) were more likely to be later in pseudotime (i.e., mature) compared with control activated macrophages (**Figure 2G**). For comparison, we analyzed differential gene expression induced by vancomycin individually in each of the six identified macrophage clusters. Vancomycin treatment was associated with upregulation in inflammation-associated KEGG pathways in most macrophage clusters (activated macrophages, metabolic A, metabolic B, and glia-like, **Supplementary Figure 8**). These data suggest that vancomycin alters macrophage maturation to retain a pro-inflammatory phenotype that is otherwise more common in newly recruited and immature macrophages.

The increase in mature activated macrophages suggested that vancomycin exposure may not just increase the number of immature macrophages present in the colon through monocyte recruitment but also influence the mature phenotype of colonic macrophages. Therefore, we assessed the impact of vancomycin exposure on differential gene expression of the combined population of muscularis and activated macrophage clusters. Control macrophages had increased expression of genes associated with anti-inflammatory microglia (*Mrc1*, *CD163*, *Ccl24*, *Retlna*), as well as ECM remodeling (*Dcn*, *Col1a1*), whereas vancomycin-exposed macrophages had increased expression of genes associated with pro-inflammatory microglia (*Ccl5*, *Il12b*, *Mmp12*) (**Figure 2H**) (62). Vancomycin induced upregulation of pro-inflammatory KEGG signaling pathways in the combined muscularis macrophage and activated macrophage cluster (**Figure 2I**).

### 3.4 Vancomycin induces the recruitment of monocyte-derived phagocytic macrophages to the neonatal colon

**Figure 3:**
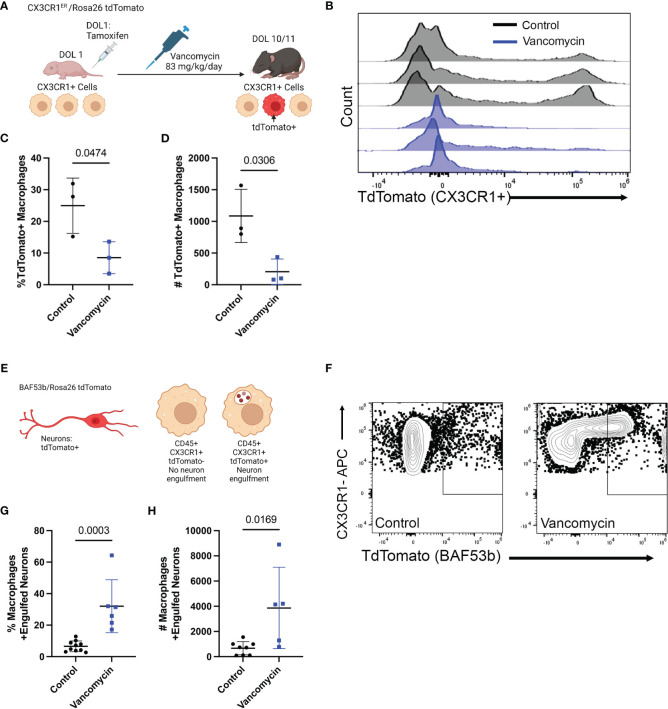
Recruitment of pro-inflammatory bone-marrow-derived macrophages drives vancomycin-induced changes in the neonatal colonic macrophage population. **(A)** Schematic demonstrating the mating and treatment protocol to label CX_3_CR1 expressing cells with tdTomato expression on DOL 1 and to assess the percentage of tdTomato+ macrophages on DOL 11. CX_3_CR1^ERT2^/tdTomato pups were treated with one dose of tamoxifen on DOL 1. Mice were then treated with vancomycin until analysis on DOL 10/11. **(B)** Histogram of tdTomato expression in CD45^+^ CX3CR1^+^ CD11b^+^ F4/80^+^ cells in control and vancomycin-treated P11 mice. Vancomycin-treated mice have significantly fewer tdTomato^+^ macrophages by both **(C)** percentage (control 24.97% ± 8.7% of macrophages, *n* = 3; vancomycin 8.54% ± 5.03% of macrophages, *n* = 3) and **(D)** absolute number of cells (control 1,086 ± 419.8 cells, vancomycin 205.7 ± 206.6 cells). **(E)** Schematic depicting tdTomato^+^ neurons as driven by the pan-neuronal BAF53b Cre. Macrophages were identified by flow cytometry, and the number of macrophages positive for tdTomato (i.e., had phagocytosed neurons) was quantified on DOL 11. **(F)** Representative flow plots from control and vancomycin-treated mice of singlet CD45^+^ CX_3_CR1^+^ cells. There are significantly more macrophages that have engulfed neurons (identified as CX_3_CR1^+^ tdTomato^+^ cells) by **(G)** percentage (control 6.57% ± 3.38% macrophages, *n* = 10; vancomycin 32.03% ± 16.8% of macrophages, *n* = 6) and **(H)** absolute number (control 668.8 ± 526.9 cells, vancomycin 3,865 ± 3,230 cells) indicating increased neuronal engulfment by macrophages in vancomycin-treated mice.

Based on gene expression, these data support that vancomycin treatment not only induces recruitment of circulating monocytes into the colon but also promotes a pro-inflammatory macrophage phenotype compared with control conditions. We next wanted to validate the changes induced by vancomycin as identified in our above RNA sequencing experiments. First, we aimed to identify the origin of the colonic macrophages in the colons of control and vancomycin-treated mice (bone-marrow-derived vs. yolk-sac-derived) (33, 34). While our RNA sequencing results suggested vancomycin-induced recruitment of bone-marrow-derived cells, our flow cytometry data did not show an expansion of F480^lo^ CD11b^hi^ cells that have previously been characterized as bone-marrow-derived tissue macrophages in other conditions (33, 51). For this investigation, we performed fate mapping experiments in which we bred CX_3_CR1^ERT2Cre^ mice to R26R^lsl^ tdTomato reporter mice to generate pups that will express tdTomato in cells expressing CX_3_CR1 at the time when treated with tamoxifen to mark their fates (**Figure 3A**). Pups were treated on DOL 1, before starting vancomycin treatment, to label CX_3_CR1 expressing macrophages at that time in life (mostly yolk-sac-derived) and their progeny as TdTomato^+^ (34). Monocytes that are later recruited to the colon and differentiate into macrophages by DOL 10/11 would be TdTomato^−^. There were significantly fewer TdTomato^+^ macrophages (CD45^+^ CX_3_CR1^+^ F4/80^+^ CD11b^+^) in the colons of vancomycin-treated mice compared with controls both by percentage and absolute numbers (**Figures 3B–D**). These data are congruent with our scRNA sequencing results suggesting that the colonic macrophages in vancomycin-treated mice are more recently recruited and driven by an influx of bone-marrow-derived monocytes rather than local proliferation of already resident yolk-sac-derived macrophages and suggest that the markers used to delineate yolk-sac-derived macrophages from non-yolk-sac-derived macrophages may not be specific in all conditions. Furthermore, we performed immunofluorescence staining for the mitotic marker Ki67 on the colonic muscularis layer from CX3CR1^GFP/WT^ heterozygous pups. There was no difference in the percentage of Ki67^+^ CX3CR1^GFP+^ cells, substantiating our observation that the increase in macrophages seen in vancomycin-treated mice was due to influx, not local proliferation of already present tissue-resident macrophages (**Supplementary Figure 9**).

Next, we assessed if vancomycin exposure increased macrophage phagocytosis as a marker of their inflammatory phenotype. Specifically, we quantified neuron engulfment by colonic macrophages as recent evidence demonstrated that muscularis macrophage prunes the postnatal ENS physiologically and vancomycin treatment reduces neuronal density in the neonatal colon (20, 32). Pan-neuronal BAF53bCre mice were crossed with TdTomato reporter mice to generate pups with TdTomato-labeled neurons. Neuron engulfment by macrophages was then quantified by flow cytometry (**Figure 3E**). Macrophages were identified as CD45^+^ CX_3_CR1^+^-positive cells. Vancomycin treatment significantly increased the percentage and absolute number of macrophages that had evidence of engulfed neurons (TdTomato positive, **Figures 3F–H**). These data validate the RNA sequencing data and demonstrate that vancomycin not only increases colonic macrophage number via recruitment of pro-inflammatory bone-marrow-derived monocytes but also results in increased neuronal phagocytosis.

### 3.5 Neonatal enteric neuron/macrophage interactions are altered by vancomycin treatment

**Figure 4:**
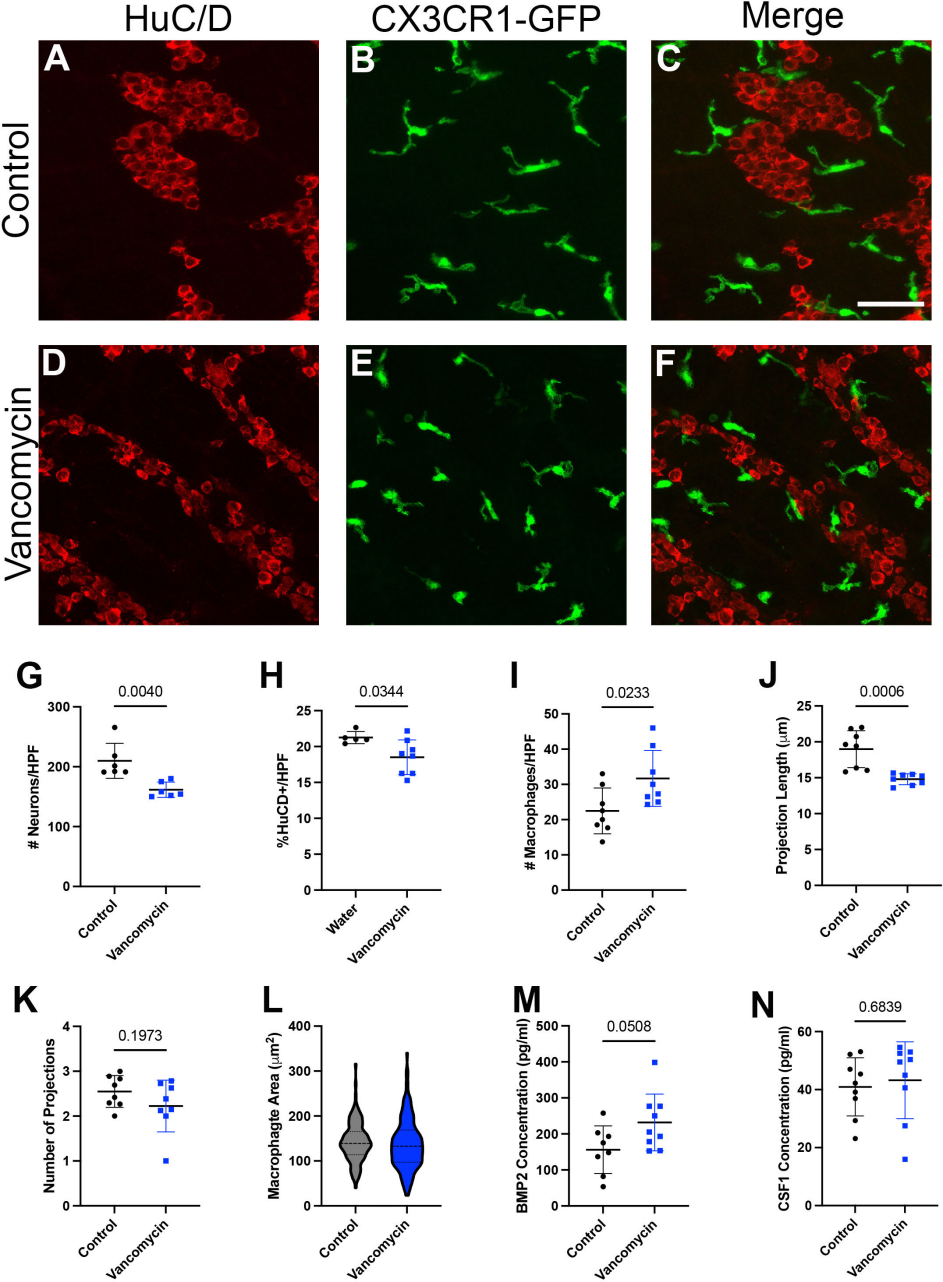
Neonatal neuron/macrophage interactions are disrupted by vancomycin treatment. Colon myenteric plexus from **(A–C)** control and **(D–F)** vancomycin-treated DOL 11 CX_3_CR1^GFP^ heterozygous mice. Scale bar = 50 µm. Vancomycin-exposed mice have reduced neuronal density in the colon myenteric plexus as measured by the number of **(G)** cell bodies (control 209.9 ± 29.23 neurons/high-power field (HPF), *n* = 6; vancomycin 161.6 ± 12.71 neurons/HPF, *n* = 6) and **(H)** field area covered by neuronal staining (control 21.25% ± 0.08%, *n* = 5; vancomycin 18.51% ± 2.42%, *n* = 8). **(I)** Vancomycin-treated mice have increased density of CX_3_CR1^GFP+^ macrophages in the muscularis (control 22.48 ± 6.48 macrophages/HPF, *n* = 8; vancomycin 31.71 ± 7.95 macrophages/HPF, *n* = 8). **(J)** The average projection length of muscularis CX_3_CR1^+^ cells was shorter in vancomycin-treated mice compared with controls (control 18.98 ± 2.58 µm, *n* = 8; vancomycin 14.8 ± 0.77 µm, *n* = 8). **(K)** There was no significant difference in the number of projections on CX_3_CR1^GFP+^ cells in the muscularis between the control and vancomycin-treated mice (control 2.55 ± 0.36 projections, *n* = 8; vancomycin 2.23 ± 0.58 projections, *n* = 8). **(L)** The area of macrophages was calculated from z-max projections of z stacks, and vancomycin-treated mice had increased variation in macrophage area compared with controls (control 142.2 ± 43.63 mm^2^, *n* = 145 macrophages; vancomycin 136.8 ± 55.5 mm^2^, *n* = 177 macrophages; Kolmogorov–Smirnov test, control *P* > 0.1, vancomycin *P* = 0.0469, non-normal distribution). ELISA was performed to measure the concentration of the growth factors **(M)** BMP2 (control 156.2 ± 66.22 pg/ml, *n* = 8; vancomycin 231.8 ± 78.83 pg/ml, *n* = 9) and **(N)** CSF1 (control 40.93 ± 10.03 pg/ml, *n* = 9; vancomycin 43.23 ± 13.24 pg/ml, *n* = 9) in total colonic lysate of control and vancomycin-treated mice pups.

Given the changes in macrophage origin and neuron engulfment due to vancomycin treatment, we next asked if the relationship between macrophages and neurons was altered in other ways by vancomycin exposure in early life. We treated CX_3_CR1^GFP/WT^ heterozygous pups with water or vancomycin from DOL 1 to 10/11 and assessed the structure of the macrophages and neurons in the muscularis layer of the colon with fluorescent microscopy (**Figures 4A–F**). Consistent with published studies, the colons of vancomycin-treated mice had significantly reduced neuronal density compared with controls as measured by neuron number and area (**Figures 4G, H**) (20). There was a significant increase in the density of macrophages in the colonic muscularis in vancomycin-treated pups compared with controls (**Figure 4I**), consistent with our flow cytometry findings (**Figures 1B–E**). The morphology of the macrophages was notably altered by vancomycin treatment. Macrophages in the colons from vancomycin-treated mice tended to have fewer and significantly shorter projections compared with macrophages in control mice (**Figures 4J, K**). These morphological characteristics are associated with activated, or inflammatory, microglia as compared with resting microglia in the central nervous system. There was increased variability in macrophage area in vancomycin-treated mice compared with controls (**Figure 4L**), consistent with the increased variability in the prevalence of macrophages in different states (i.e., resting vs. activated) (63).

Given the role of the microbiota in establishing the symbiotic growth factor secretion required for macrophage/enteric neuron crosstalk in adult mice, we measured CSF1 and BMP2 levels in the colons of control and vancomycin-exposed mice using ELISA. While there was no difference in CSF1 levels between the two groups, colonic lysates from vancomycin-treated animals had higher levels of BMP2 (**Figures 4M, N**). Notably, many cell types in the colon are capable of producing BMP2, and previous studies have established that macrophages do not become the primary source of BMP2 until postweaning ages (30). To determine how vancomycin treatment altered BMP2 expression in neonatal colonic macrophages, we returned to our RNA sequencing data. BMP2 was expressed by all six macrophage clusters identified (**Supplementary Figure 10A**). In contrast to our protein expression data in whole colonic lysate, there was decreased BMP2 gene expression in the pooled six macrophage clusters treated with vancomycin (**Supplementary Figure 10B**). These data suggest that the increased BMP2 expression seen on the protein level in vancomycin-treated colons is due to the upregulation of expression in other cell types and not macrophages.

### 3.6 Inhibition of recruitment of monocyte-derived macrophages prevents vancomycin-induced effects on the enteric nervous system

**Figure 5:**
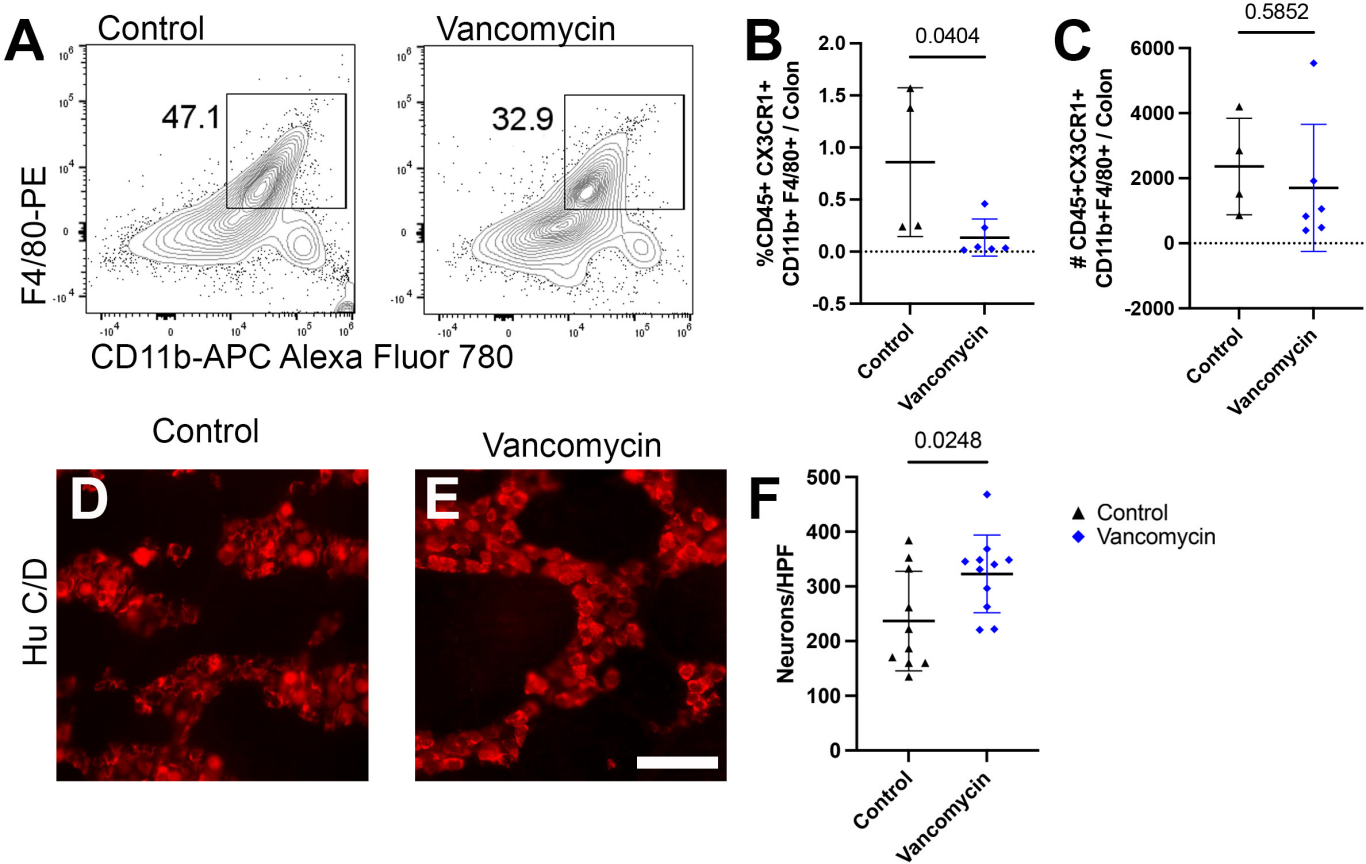
Vancomycin effects on neuronal or macrophage density are abrogated in mice deficient in bone-marrow-derived macrophage recruitment. **(A)** Representative density plots of singlet CD45^+^ CX_3_CR1^+^ CD11b^+^ F4/80+ cells from control and vancomycin-treated CCR2-deficient mice, respectively. There was a decrease in CD45^+^ CX_3_CR1^+^ CD11b^+^ F4/80^+^ cells in vancomycin-treated CCR2-deficient mice when compared with controls by **(B)** percentage (control 0.86% ± 0.71% of total cells, *n* = 4; vancomycin 0.13% ± 0.18% of total cells, *n* = 6), but there was no change in the **(C)** absolute number of cells (control 2,360 ± 1,483 cells, *n* = 4; vancomycin 1,702 ± 1,955 cells, *n* = 6). **(D, E)** Colonic myenteric plexus stained for neurons with HuC/D in CCR2-deficient control and vancomycin-treated pups. Scale bar = 50 µm. **(F)** Quantification of neurons/HPF demonstrated an increase in neuronal density in vancomycin-treated CCR2-deficient mice compared with controls (control 236.7 ± 90.9 neurons/HPF, *n* = 10; vancomycin 322.9 ± 71 neurons/HPF, *n* = 11).

Finally, vancomycin appears to impact the colonic macrophage population both via increased recruitment of bone-marrow-derived macrophages as well as inducement of differentiation into a more inflammatory macrophage. However, it is unclear if only the newly recruited macrophages are subject to be induced pro-inflammatory phenotype or if vancomycin treatment influences existent tissue-resident colonic macrophages. To decouple these observations, we utilized mice deficient in the CCR2 receptor, which is vital for the recruitment of bone-marrow-derived circulating monocytes into the colon, resulting in reduced numbers of bone-marrow-derived macrophages in the neonatal period (51, 64). Using flow cytometry, we compared the colonic macrophages between control and vancomycin-treated CCR2-deficient pups. CCR2-deficient mice treated with vancomycin had a decrease in the proportion of macrophages (CD45^+^ CX_3_CR1^+^ CD11b^+^ F4/80^+^) of total colonic cells and no change in the absolute number of macrophages when compared with controls (**Figures 5A–C**). This is in contrast to the increase in macrophages seen in vancomycin-treated wild-type mice (**Figure 1**), indicating that the vancomycin-induced increase in colonic macrophages was CCR2-dependent. We next assessed if the vancomycin-induced changes in enteric neuron density were abrogated in CCR2-deficient mice. Compared with CCR2-deficient controls, vancomycin-exposed CCR2-deficient mice had an unexpected increase in colonic myenteric neuron density (**Figures 5D–F**). These data suggest that the vancomycin-induced changes in colonic macrophage number and enteric nervous system development are driven by a CCR2-dependent influx of newly recruited bone-marrow-derived macrophages.

## 4 Discussion

Vancomycin is the fourth most used drug in the neonatal intensive care unit (NICU) (2). While antibiotics given to neonates in the NICU can be lifesaving, they are also associated with a short-term increased risk of LOS and NEC (11). Early-life antibiotic exposure is also associated with a longer-term increased risk of some immune pathologies which are believed to occur due to disruption of the microbiota during a critical time in early life (13). Prior studies have shown that a short course of vancomycin in preweaning mice results in a decreased density of enteric neurons, yet how vancomycin mediates this effect is unclear (20). Here, we show that the effects of a short course of vancomycin in the preweaning period are, in part, mediated by CCR2-dependent recruitment of monocytes/macrophages into the colon. Furthermore, vancomycin altered the phenotype of the colonic macrophages to become more inflammatory, resulting in an increased phagocytosis of colonic myenteric neurons. Thus, the reduced colonic myenteric neuron density induced by vancomycin in early life is, in part, due to changes in the colonic macrophage population. These changes were not seen in postweaning mice given similar courses of vancomycin, indicating that these alterations are unique to the preweaning period.

Multiple factors may explain why antibiotic treatment during this preweaning period uniquely alters long-term outcomes. During this period, the ENS, gut microbiome, and immune system are undergoing dramatic changes as they develop into more stable entities. Alterations in the gut microbiome during this period have been demonstrated to have durable effects on the ENS and gut immune system development (20, 21). In particular, macrophages have been found to closely interact with the ENS. During the neonatal period, the colonic macrophage population is undergoing significant changes. Yolk-sac-derived macrophages are being replaced or diluted out by bone-marrow-derived macrophages (32–34). Notably, the macrophages interacting with the ENS in adult mice are overwhelmingly long-lived yolk-sac-derived macrophages. We also know that microbes and microbial ligands can have significant effects on macrophage phenotype. Therefore, it is likely that disruption of the microbiota alters colonic macrophage development during this preweaning period, which then can affect the ENS development. Alternatively, microbial ligands might directly interact with the ENS and potentially affect ENS development independent of the effects on macrophages. In support of the former possibility, we observed that CCR2-deficient mice, which fail to recruit bone-marrow-derived macrophages to the colon, did not have reduced colonic ENS neuronal density when given vancomycin during this preweaning period. This is consistent with these effects being mediated by macrophages. Interestingly, in a previous work, we and others have found that preweaning antibiotics do not significantly affect the ENS in the small intestine (20). This regional difference could be due to the increased density of the microbiota in the colon compared with the small intestine, as well as the proximal to distal timing of ENS development after birth. ENS development in the small intestine in germ-free mice is less affected compared with the colon. This indicates that ENS development in the small intestine is less dependent upon the microbiota, potentially supporting a preferential role for microbes and/or microbial metabolites as a driving factor for colonic versus small intestine ENS development (19).

In adults, the interaction of macrophages and enteric neurons is vital for homeostasis and is known to be dependent upon the gut microbiota (27, 65). However, little is known about how these interactions develop in early life or how they are affected by alterations in the gut microbiota. Alterations in the development of any one of these components will have implications in the development of the other two. Several studies exploring the impact of the microbiota (i.e., germ-free mice and ampicillin treatment) on the adult ENS have identified decreased levels of microbially derived serotonin as the driving factor for the observed ENS hypodensity (20, 66). Notably, no decrease in either ENS density or mucosal serotonin levels was observed in postnatal mice treated with vancomycin (52). In neonatal mice treated with vancomycin, supplementation with the serotonin precursor 5-HTP rescued the colonic hypoganglia. The microbiota serves as the source for a plethora of neurotransmitters in the preweaning mouse. Specifically, bacteria in the *Rodentibacter* genus appear to play a significant role in the production of serotonin in the neonatal mouse intestine (50). Notably, *Proteobacteria* phylum (of which the *Rodentibacte*r genus is part of) abundance was decreased in previous studies with neonatal vancomycin treatment (20). While we did not appreciate this taxonomic change in our microbial profiling, we did observe a significant decrease in luminal serotonin levels in vancomycin-treated neonatal mice. Given that neuronal signaling can induce an anti-inflammatory phenotype in colonic macrophages, an altered availability of luminal neurotransmitters may be enough to induce the changes we observe in vancomycin-treated colonic macrophages (31, 67). These data also indicate that V4 16S RNA sequencing may not be granular enough to identify functional changes in the gut microbiota of neonatal mice particularly due to the overall low diversity of bacteria that can be classified at the taxonomic level with this method. Alternatively, neonatal antibiotic treatment may be affecting specific bacterial strain abundance and/or gene expression in bacteria to disrupt neonatal intestinal homeostasis, as evidenced by our observed changes in serotonin, a bacterial metabolite. More in-depth analysis will be required going forward to characterize the impact of antibiotics on the neonatal microbiota beyond higher-level taxonomic evaluation.

A recent work identified a role for the muscularis macrophages in pruning the ENS during a critical window in early postnatal development (32). Our study shows how vancomycin treatment expands and skews the colonic macrophage phenotype toward a pro-inflammatory signature and subsequently increases engulfment in enteric neurons. This appears to be an effect that is restricted to the neonatal period, as vancomycin did not expand the macrophage population in adult mice. The critical period associated with macrophage-dependent ENS pruning matches with the observed effects of vancomycin treatment on colonic ENS density. Vancomycin treatment in the neonatal versus postweaning period has distinct effects on the ENS. Neonatal treatment is associated with reduced neuronal density, while postnatal treatment has no effect on neuronal density (20, 52). Notably, both neonatal and postweaning vancomycin treatments are associated with alterations in the subtype specification of enteric neurons, but the changes induced are different depending on the age at which the exposure occurs. Consistent with the window of opportunity hypothesis, neonatal vancomycin treatment confers changes to the structure and function of the ENS that persist through adulthood. As the ENS interacts with the microbiota directly via constitutive expression of Toll-like receptors, it is possible that the changes in subtype specification seen in the ENS after antibiotic exposure at any age may be cell autonomous (21). The lack of change in neuronal density in mice treated with vancomycin after weaning supports the neonatal time point as a critical window in which ENS pruning could be impacted by a change in muscularis macrophage phenotype. These studies, and a recent work demonstrating a preweaning critical period in which muscularis macrophages prune the postnatal ENS, support our findings that changes in macrophage phenotype due to neonatal dysbiosis impact ENS development secondary to increased neuron engulfment (32).

Our data demonstrate that vancomycin induces reduced enteric neuron density due to neuronal engulfment by an altered colonic macrophage population influenced by the influx of pro-inflammatory bone-marrow-derived macrophages. However, the catalyst for this macrophage recruitment is still unknown. Studies in adult mice have shown that in certain contexts, enteric glia will produce CSF1, the macrophage growth factor canonically thought to be produced by enteric neurons in intestinal homeostasis (68, 69). Notably, we did not observe an increase in CSF1 in the colonic tissue of our vancomycin-treated mice. This is consistent with previous studies which have found that the ENS is not the primary intestinal source for CSF1 until postweaning (30). Furthermore, the impetus for the physiologic recruitment of bone-marrow-derived macrophages over the course of the preweaning is still unknown. A microbial source has been hypothesized, but despite the expansion of embryonic-derived macrophages, there is not a dearth of bone-marrow-derived macrophages in germ-free mice. Notably, the adult enteric and peripheral nervous system express CCL2, the canonical ligand for CCR2 (26). As the effect of vancomycin on the neonatal macrophage and ENS is CCR2-dependent, further investigation into differential expression of CCL2 in control and vancomycin-treated colons, specifically in ENS cells, is warranted. Additionally, other ligands have been suggested as potential candidates to promote monocyte tissue recruitment and/or differentiation, including transforming growth factor β (TGF-β), especially in the neonatal bowel (64, 70, 71). Still, this is unlikely given the CCR2-dependent nature of the vancomycin phenotype. TGF-β was recently shown to be an important mechanism of the crosstalk between enteric neurons and macrophages in neonatal mice and represents an additional vehicle for further investigation (32). Compared with adults, intestinal inflammation in neonates is marked by an influx of macrophages as observed in necrotizing enterocolitis, a lethal inflammatory disease in premature neonates (72, 73). Vancomycin-mediated dysbiosis may be inducing intestinal injury leading to macrophage infiltration and downstream effects on the ENS.

In conclusion, in this study, we demonstrate that a clinically relevant antibiotic exposure (both in the choice of antibiotic and the timing and duration of treatment) is sufficient to alter the neonatal microbiota and disrupt macrophage homeostasis in the colon. Despite greater effects on the microbiota, vancomycin treatment of adult mice does not elicit the same changes in macrophage number and phenotype as observed in neonatal mice. Single-cell RNA sequencing of CX_3_CR1^+^ macrophages in the neonatal mouse colon revealed that vancomycin exposure increased the prevalence of macrophages with a pro-inflammatory phenotype in bone-marrow-derived macrophages. We were able to validate these changes via morphological analysis and lineage tracing experiments. We demonstrated that these observed changes in neuronal and macrophage density induced by vancomycin treatment are driven by the recruitment of macrophages and do not occur in CCR2 KO mice which have impaired monocyte migration into the colon. Further investigation is necessary to elucidate the microbial and molecular signaling molecules mediating these vancomycin-induced changes in order to identify novel strategies to protect intestinal homeostasis in antibiotic-treated neonates with the goal of reducing morbidity and mortality associated with early-life antibiotic exposure in premature neonates.

## Data availability statement

The datasets presented in this study can be found in online repositories. The names of the repository/repositories and accession number(s) can be found below: https://www.ncbi.nlm.nih.gov/, GSE74131.

## Ethics statement

The animal study was approved by the Institutional Animal Care and Use Committee at Washington University School of Medicine. The study was conducted in accordance with the local legislation and institutional requirements.

## Author contributions

ES: Conceptualization, Formal Analysis, Funding acquisition, Investigation, Methodology, Visualization, Writing – original draft, Writing – review & editing. EJ: Formal Analysis, Investigation, Writing – original draft, Writing – review & editing. AF: Investigation, Writing – review & editing. SU: Formal Analysis, Resources, Visualization, Writing – original draft, Writing – review & editing. BR: Formal Analysis, Writing – review & editing. SG: Investigation, Writing – review & editing. BB: Investigation, Writing – review & editing. VJ: Investigation, Writing – review & editing. MK: Investigation, Writing – review & editing. DK: Formal Analysis, Writing – review & editing. JP: Investigation, Writing – review & editing. KM: Investigation, Project administration, Supervision, Writing – review & editing. RN: Conceptualization, Funding acquisition, Project administration, Resources, Supervision, Writing – original draft, Writing – review & editing.
