# Supplementary material for: Vancomycin-induced gut microbial dysbiosis alters enteric neuron–macrophage interactions during a critical period of postnatal development
Source: Front Immunol. 2023 Oct 12;14:1268909. doi: 10.3389/fimmu.2023.1268909 (PMC10602895; doi:10.3389/fimmu.2023.1268909)
Supplement: Supplementary file 1 [file DataSheet_1.docx]

Supplementary Material

Vancomycin Induced Gut Microbial Dysbiosis Alters Enteric Neuron-Macrophage Interactions During a Critical Period of Postnatal Development

Ellen Merrick Schill^1,2,*^, Elisabeth L. Joyce^1^, Alexandria N. Floyd^1^, Sreeram Udayan^1^, Brigida Rusconi^3^, Shreya Gaddipati^1^, Bibiana E. Barrios^1^, Vini John^1^, Mitchell E. Kaye^1^, Devesha H. Kulkarni^1^, Jocelyn T. Pauta^1^, Keely G. McDonald^1^, Rodney D. Newberry^1,*^

^1^Division of Gastroenterology, Department of Medicine, ^2^Divsion of Newborn Medicine, and ^3^Division of Gastroenterology, Hepatology & Nutrition, Department of Pediatrics Washington University School of Medicine, St. Louis, MO 63110

*Correspondence: EMS: [merricke@wustl.edu](mailto:merricke@wustl.edu), RDN: [rnewberry@wustl.edu](mailto:rnewberry@wustl.edu)

## Supplementary Figures

**Supplementary Figure 1: Vancomycin treatment significantly alters alpha and beta diversity in adult, but not neonatal mice.** (A) Alpha diversity (Control 2.15±0.6 n=4 litters, Vancomycin 2.49±1.1 n=3 litters) measured by Shannon entropy and (B) beta diversity measured by Bray-Curtis was not different in control and vancomycin neonatal mice. (C) Alpha diversity (Control 5.18±0.48 n=4, Vancomycin 3.58±0.01, n=4) measured by Shannon entropy and (D) beta diversity measured by Bray-Curtis was significant different in adult mice.

**Supplementary Figure 2: Gating strategy for CD45^+^ CX3CR1^+^ CD11b^+^ F4/80^+^ colonic macrophages.** (A) Gating strategy to identify live, CD45^+^ CX3CR1^+^ CD11b^+^ F4/80**^+^** cells – defined as colonic macrophages. (B) Full minus one staining for CX3CR1, CD11b and F4/80. Gating for CD11b and F4/80 is based on the inclusion of the spectrum of colonic macrophages present at DOL11 which includes CD11b^+^ F4/80^low/-^ cells [33].

**Supplementary Figure 3: Vancomycin treatment did not significantly impact the percentage of F4/80^hi^ macrophages in neonatal mice.** Representative contour plots from (A) control and (B) vancomycin treated colonic macrophages (CD45^+^ CX3CR1^+^ CD11b^+^ F480^+^) gated on F4/80^hi^ consistent with yolk-sac-derived macrophage signature [33, 51]. (C) There was no significant difference in the percentage of colonic macrophages identified as F4/80^hi^ between control and vancomycin treatment.

**Supplementary Figure 4: Gating strategy to select colonic macrohages to isolate for single cell RNA sequencing by fluorescent activated cell sorting.** Single cell suspensions of colon were generated from control and vancomycin treated CX_3_CR1^GFP^ heterozygous pups. All pups from a single litter were pooled for sorting. Cells were selected for Live (top left), CD45^+^ (top right), CD11c^lo/-^ (bottom left), and CXC3CR1^GFP+^ (Bottom right). Cells were collected in serum enriched media and submitted for sequencing.

**Supplementary Figure 5:** **Original 11 Clusters identified by unbiased algorithm from single cell RNA sequencing of sorted CX_3_CR1^+^ cells.** UMAP showing down-sampled, pooled counts from control and vancomycin treated cells color coded by cluster number. Table below showing how non-macrophage clusters were labeled as well as biomarkers and percent abundance in control and vancomycin treated cohorts of each cluster.

**Supplementary Figure 6: KEGG Pathways enriched in each of the six macrophage clusters as elucidated via biomarker identification.** Biomarkers from each cluster were analyzed with WEBGESTALT [54] and the most significantly upregulated pathways (with FDR<0.05) graphed by enrichment score.

**Supplementary Figure 7: Muscularis Macrophage and Activated Macrophage clusters do not appear to differ by tissue (muscularis or mucosal) tissue of origin, but activated macrophages have upregulation of inflammatory signaling pathways.** (A) Volcano plot of differentially regulated genes between the activated (left) and muscularis (right) macrophage clusters. Gene lists comparing expression in macrophages isolated from muscularis tissue versus mucosal tissue (from published scRNA sequencing in adult mice [57]) were compared to our muscularis and activated macrophages and neither cluster was found to be upregulated in muscularis vs. mucosal genes. The activated cluster had more upregulated genes compared to the muscularis cluster and more genes in common with both the muscularis and mucosal macrophages in adult mice. (B) Genes differentially expressed between the activated (left) and muscularis (right) cluster were identified by GSA and analyzed via WEBGESTALT [54]. Activated macrophages had enrichment in pro-inflammatory signaling pathways compared to the muscularis macrophages.

**Supplementary Figure 8: Vancomycin treatment upregulates gene expression in inflammatory signaling pathways in most colonic macrophage subsets.** Genes differentially expressed between control and vancomycin treated mice in each macrophage cluster were identified by GSA (FDR of <0.05 and fold change of less than -2 or greater than 2). There were 66 differentially regulated genes between control and vancomycin in the muscularis macrophage cluster, 2657 in the activated macrophage cluster, 148 in the metabolic A cluster, 1545 in the metabolic B cluster, 139 in the Phagocytosed-ECM cluster, and 734 in the glia-like cluster. KEGG pathways associated with immune activation and inflammation were noted in 4 clusters (activated macrophages, metabolic A and B, and glia-like). Vancomycin did not significantly alter pathways in the Phagocytosed-ECM cluster.

**Supplementary Figure 9: Vancomycin treatment does not impact colonic macrophage proliferation.**  Colonic muscularis layer from (A-C) control or (D-F) vancomycin treated DOL10/11 CX3CR1^GFP+/-^ mice were stained for the proliferation marker Ki67. (G) There was no difference in the percentage of proliferating macrophages (Ki67^+^ CX3CR1^GFP+^ cells) between the two conditions. Scale bar = 20μm

**Supplementary Figure 10: All macrophage clusters express BMP2, but vancomycin decreases overall BMP2 gene expression in macrophages.** (A) Dot plot showing expression (by LSMEAN) of BMP2 in each macrophage cluster (control and vancomycin pooled). (B) Vancomycin decreased BMP2 gene expression by macrophages (all 6 clusters pooled), but this did not reach statistical significance (Control LSMean 53.51, Vancomycin LSMean 20.05, p=0.24, FDR = 0.29).

## Supplementary Table

**Supplementary Table 1. Antibodies used for Flow Cytometry and Immunofluoresence**

| ***Flow Cytometry Antibodies*** | ***Conjugate*** | ***Working Dilution*** | ***Vendor*** | ***Catalog #*** |
| --- | --- | --- | --- | --- |
| anti mouse CD11b | APC eFluor 780 | 1:100 | ebioscience | 47-0112 |
| anti mouse F4/80 | PE | 1:100 | Invitrogen | 12-4801 |
| anti mouse CD11c | AF 700 | 1:100 | ebioscience | 56-0114 |
| anti mouse CD45 | PE-Cy7 | 1:100 | eBioscience | 25-0451 |
| anti mouse F4/80 | PerCP-Cy5.5 | 1:100 | eBioscience | 45-4801 |
| anti mouse CD11b | ef450 | 1:100 | ebioscience | 48-0112 |
| anti mouse CX3CR1 | APC | 1:100 | Biolegend | 149004 |
| ***Immunofluoresence Antibodies*** | ***Conjugate*** | ***Working Dilution*** | ***Vendor*** | ***Catalog #*** |
| ANNA-1 |  |  | Kind gift from Dr. Verna Lennon |  |
| Rabbit anti HuC/HuD | AF647 | 1:500 | Abcam | ab237235 |
| Rabbit anti Ki67 |  | 1:250 | ThermoFisher | MA5-14520 |
| Donkey anti Rabbit | AF 594 | 1:500 | ThermoFisher | A-21207 |
| Donkey anti Human | AF 647 | 1:500 | Jackson ImmunoResearch | 709605149 |

**Supplementary Table 2. ANCOM Statistical Analysis for Adult Control vs. Vancomycin Microbiota**

**Supplementary Table 3. Biomarkers for 11 Original clusters of CX3CR1+ scRNA Sequencing**

**Supplementary Table 4. Biomarkers for 6 Macrophage clusters from scRNA Sequencing**
