# Supplementary figures and images for: Vancomycin-induced gut microbial dysbiosis alters enteric neuron–macrophage interactions during a critical period of postnatal development
Source: Front Immunol. 2023 Oct 12;14:1268909. doi: 10.3389/fimmu.2023.1268909 (PMC10602895; doi:10.3389/fimmu.2023.1268909)

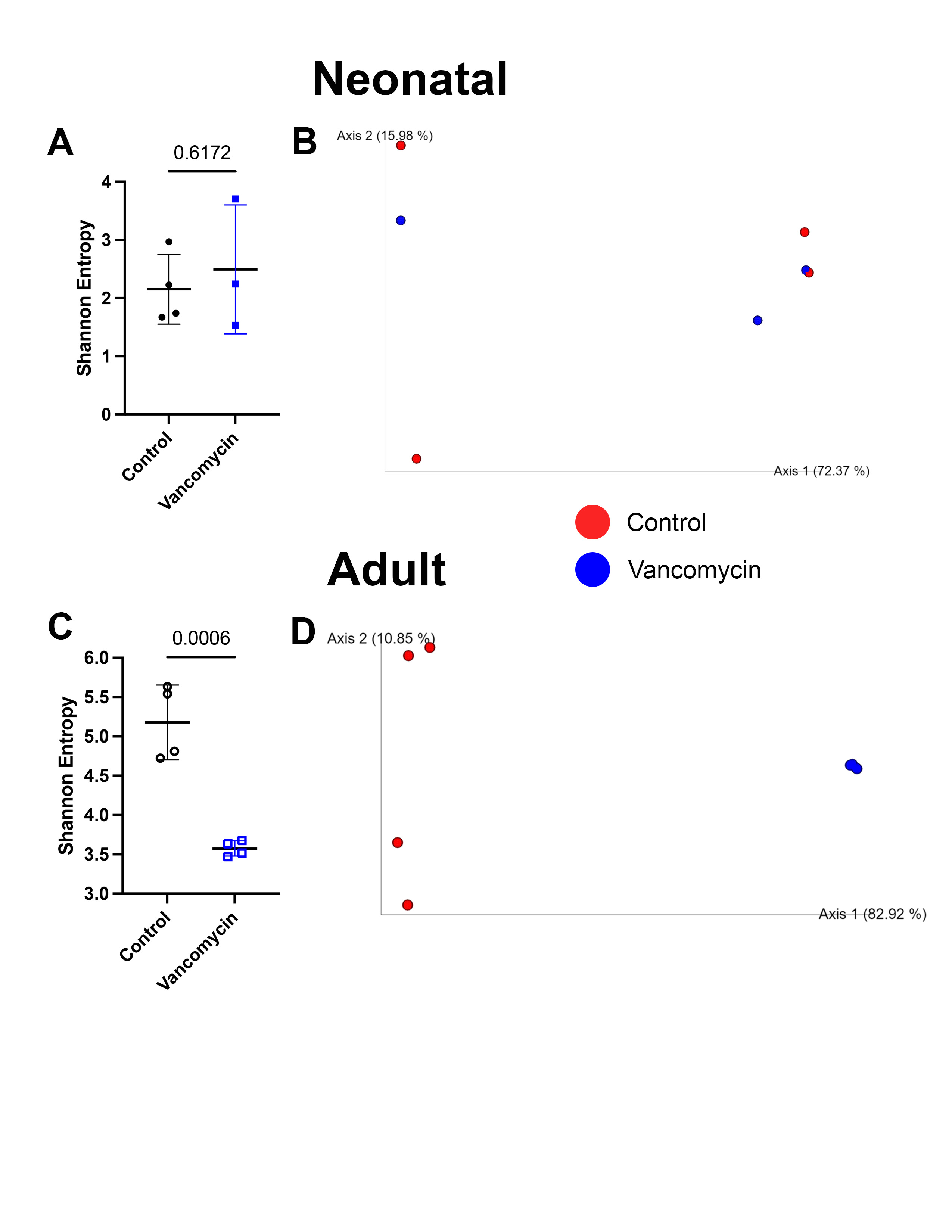

Supplement: Supplementary file 2 [file Image_1.jpeg]

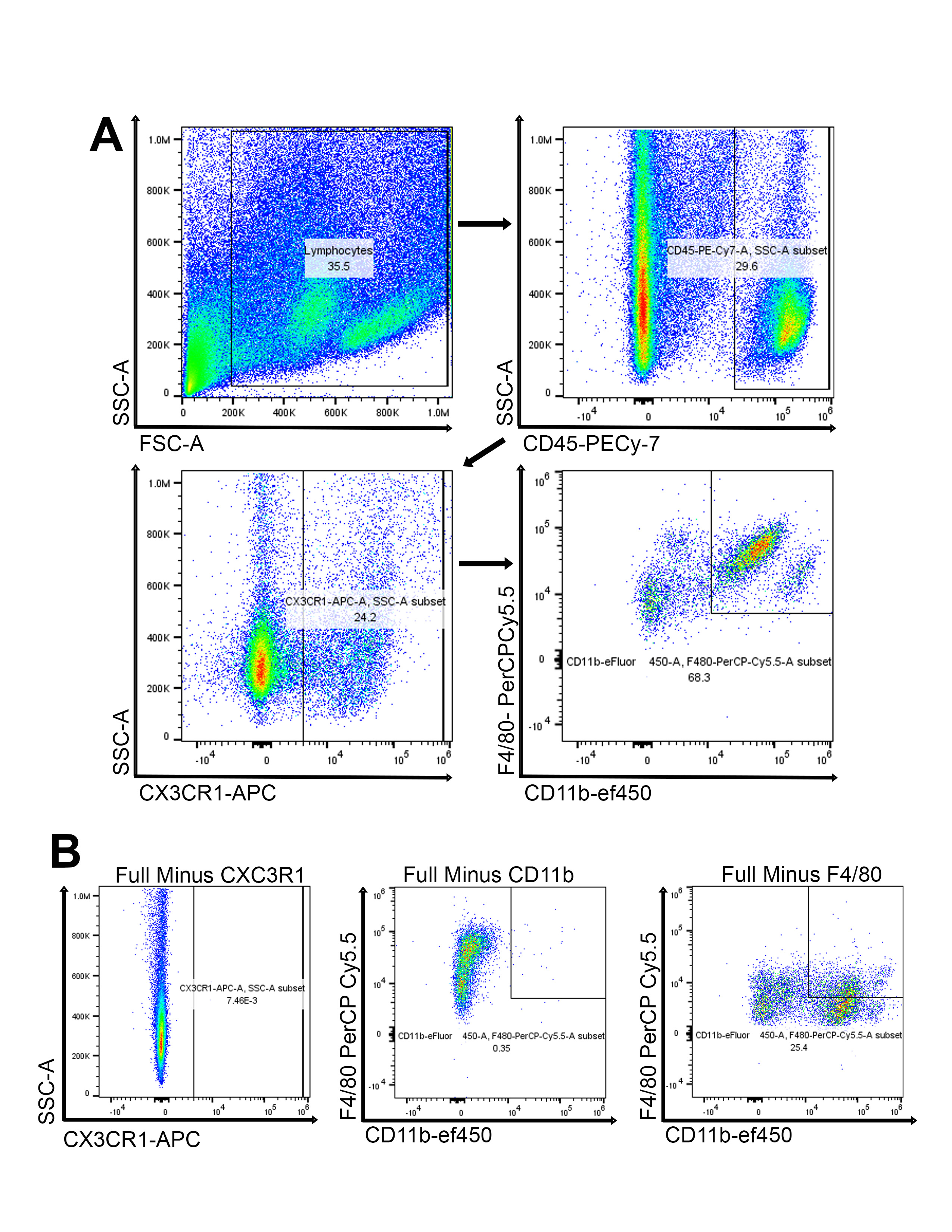

Supplement: Supplementary file 3 [file Image_2.jpeg]

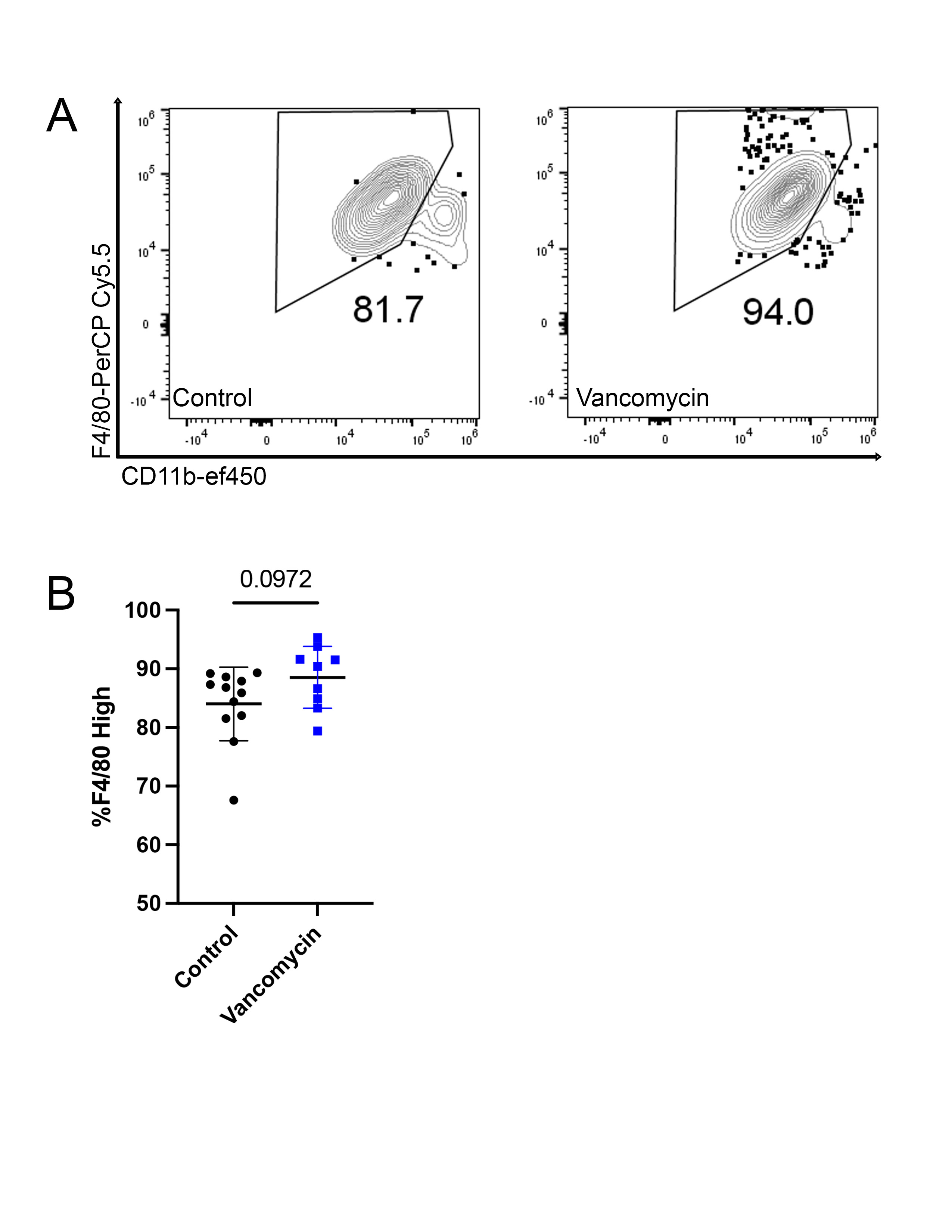

Supplement: Supplementary file 4 [file Image_3.jpeg]

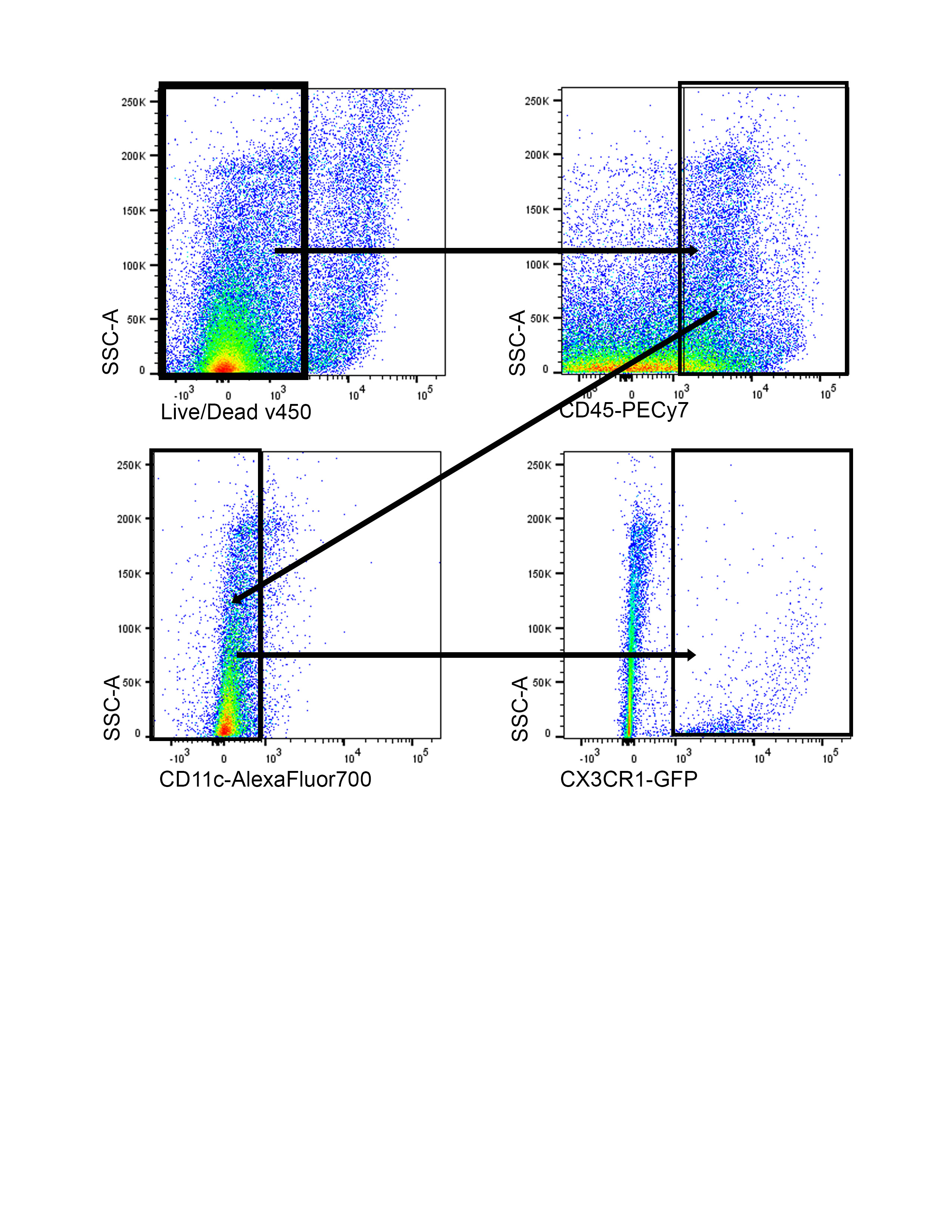

Supplement: Supplementary file 5 [file Image_4.jpeg]

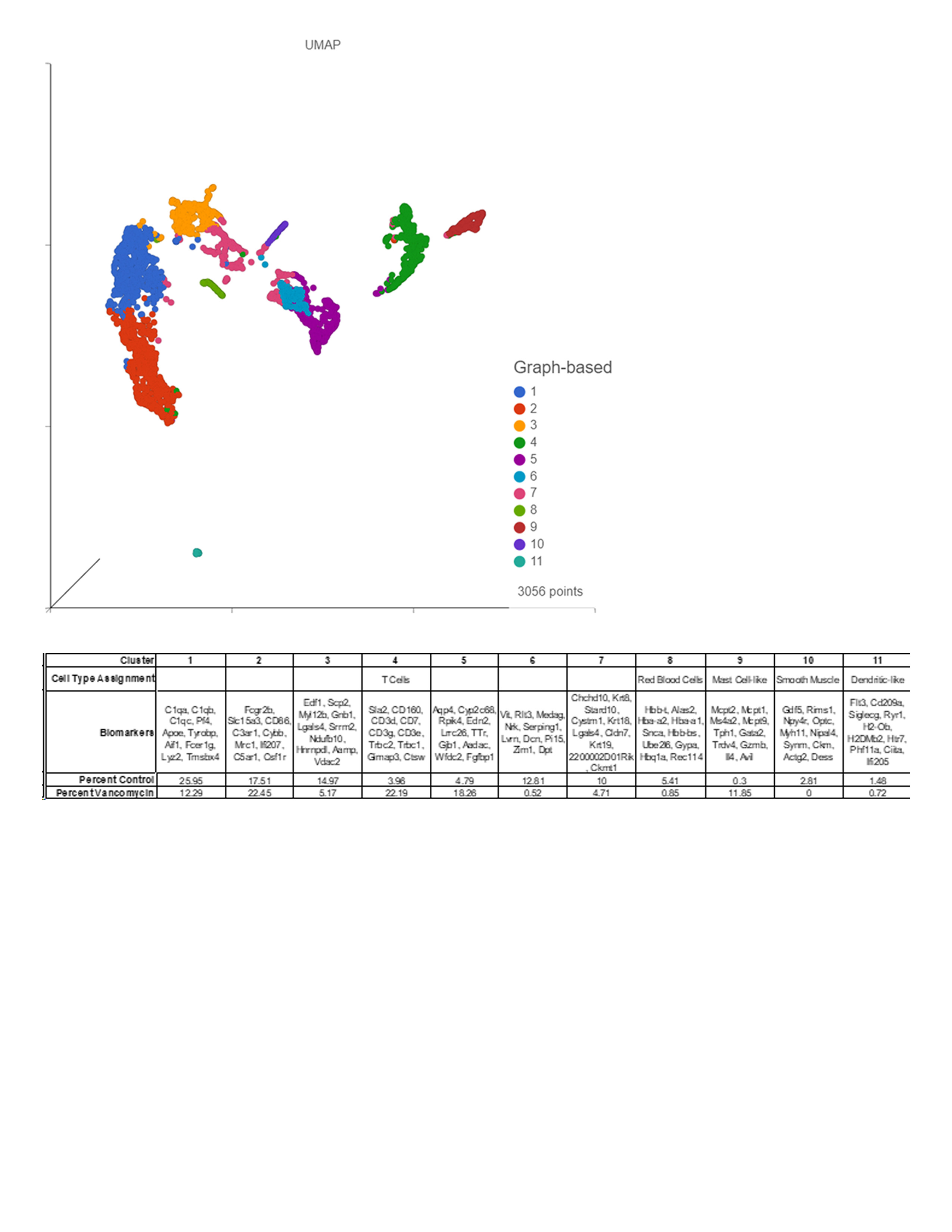

Supplement: Supplementary file 6 [file Image_5.jpeg]

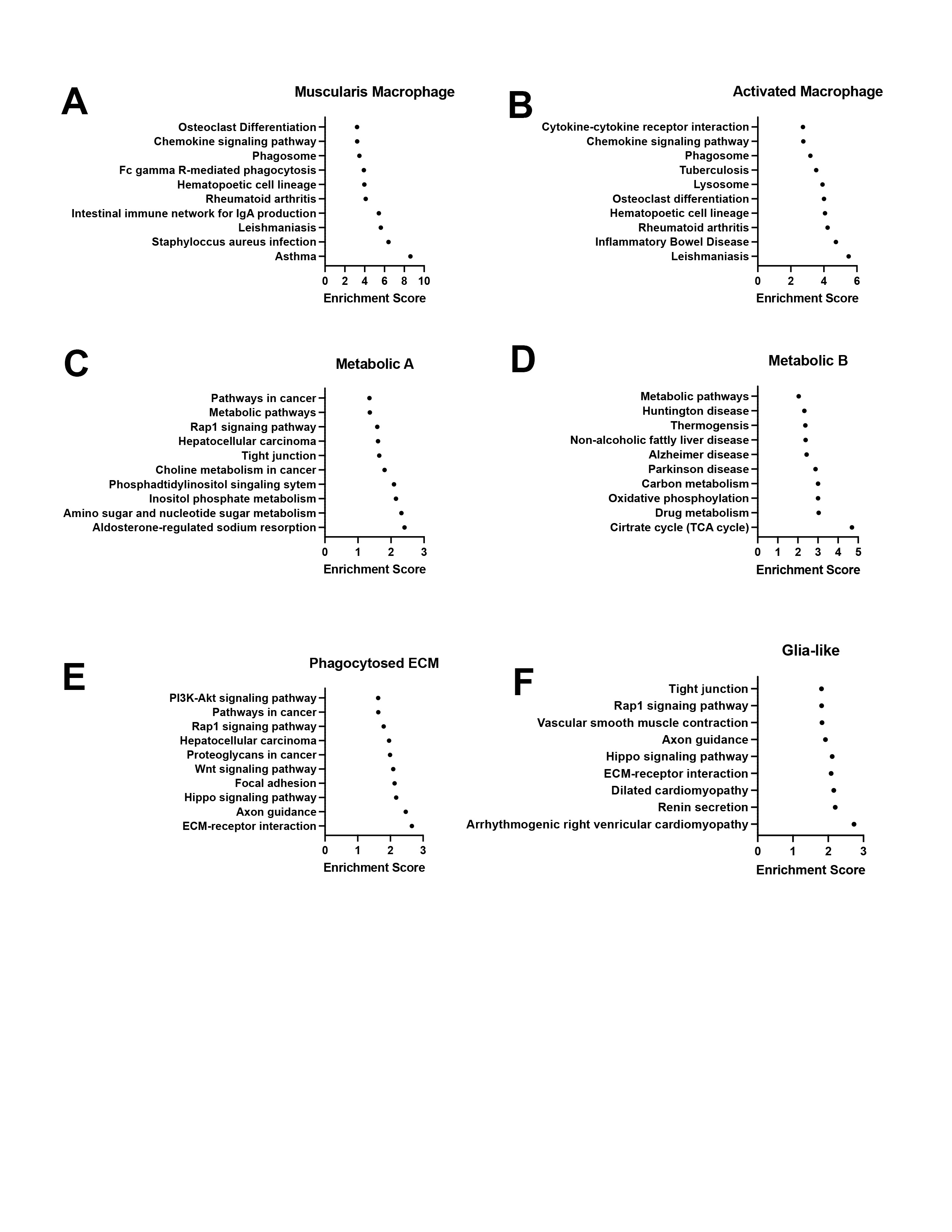

Supplement: Supplementary file 7 [file Image_6.jpeg]

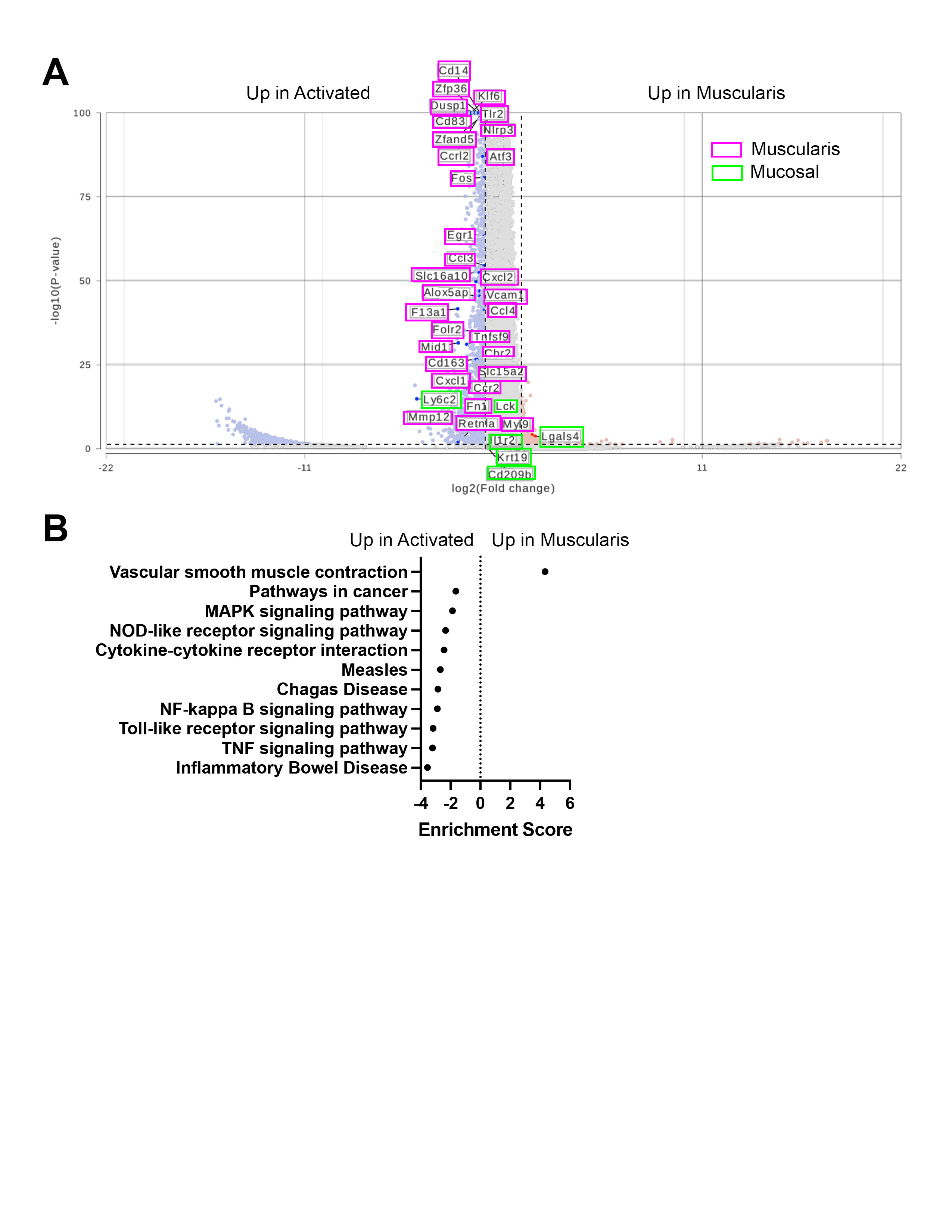

Supplement: Supplementary file 8 [file Image_7.jpeg]

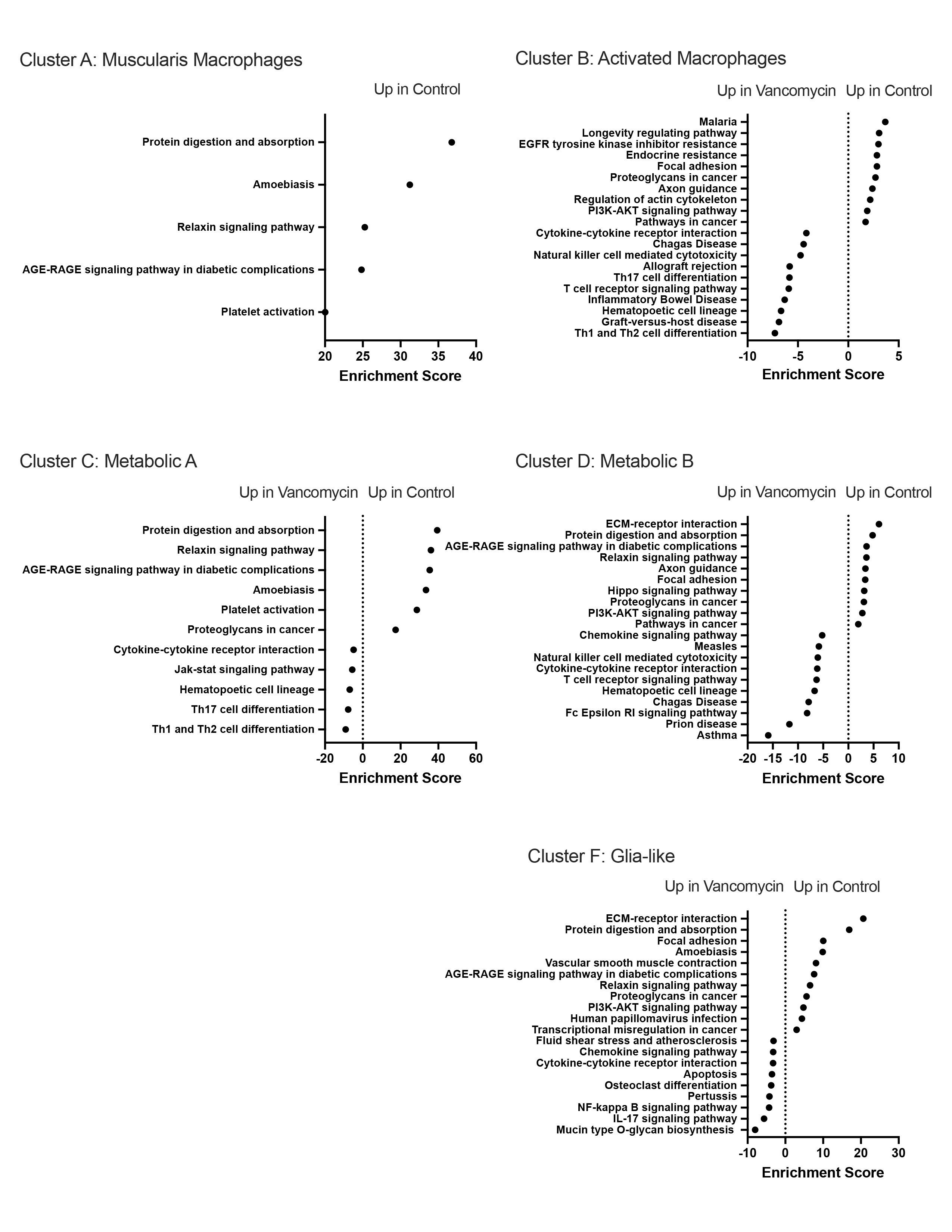

Supplement: Supplementary file 9 [file Image_8.jpeg]

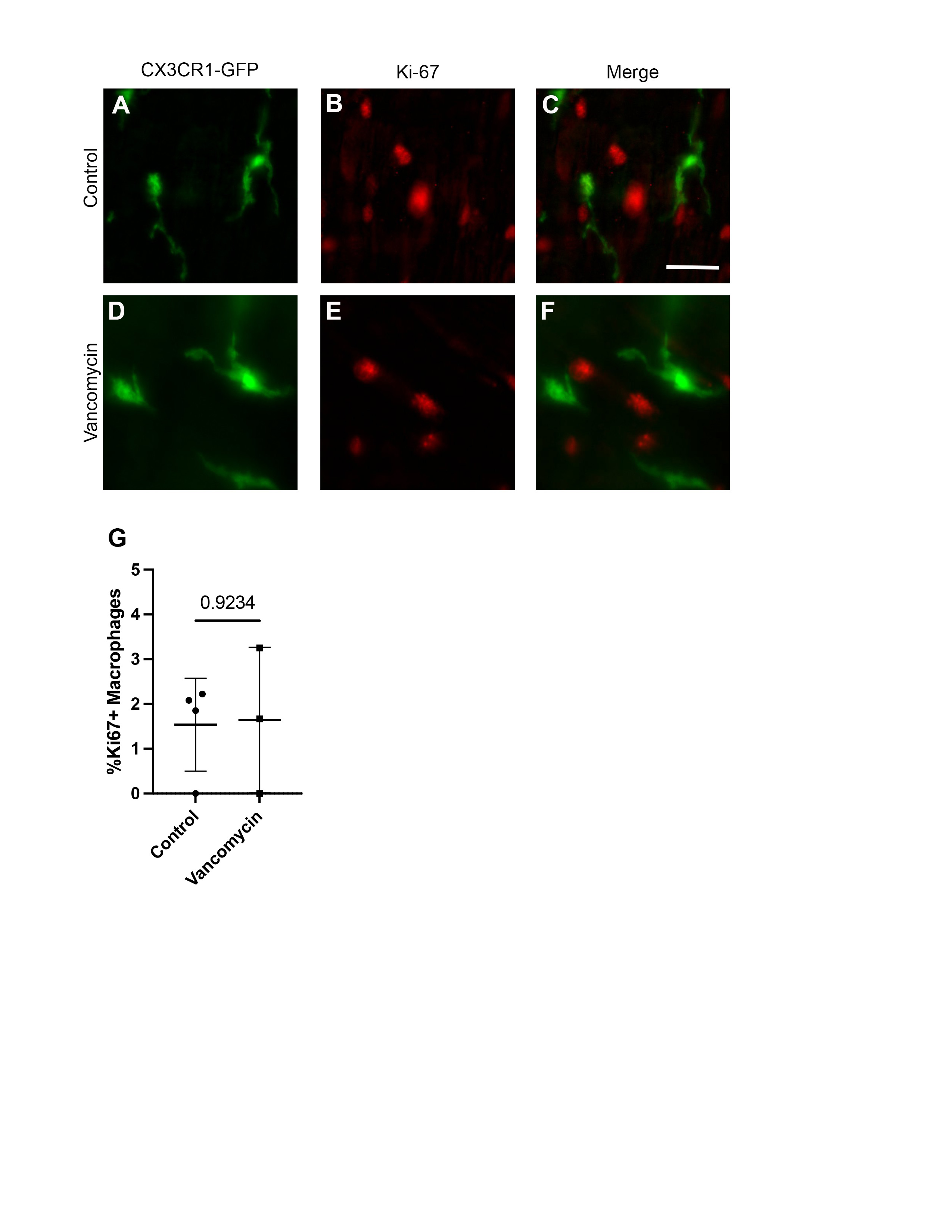

Supplement: Supplementary file 10 [file Image_9.jpeg]

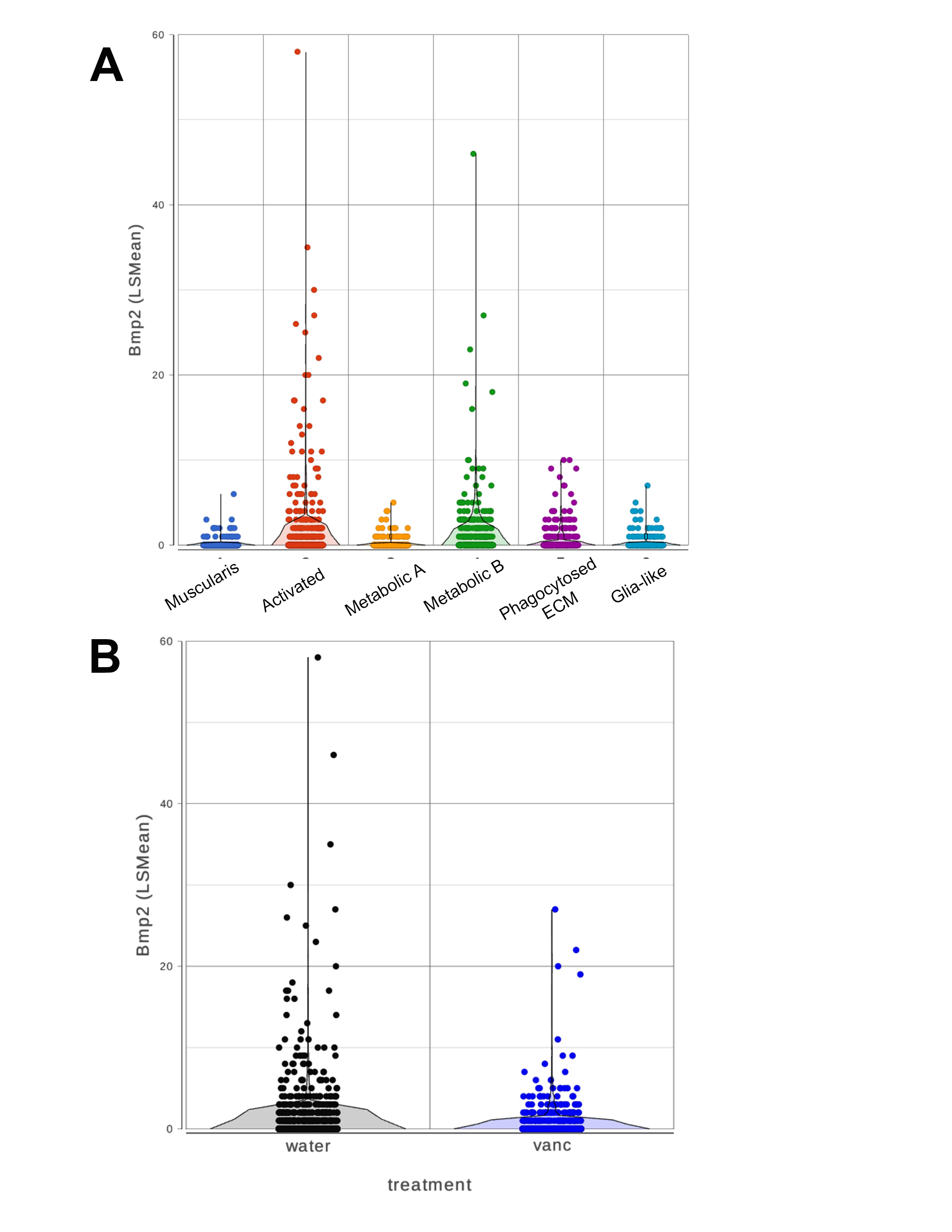

Supplement: Supplementary file 11 [file Image_10.jpeg]
